# The Prognostic Significance of Selected HLA Alleles on Prostate Cancer Outcome

**DOI:** 10.3390/ijms241914454

**Published:** 2023-09-22

**Authors:** Savvas Stokidis, Constantin N. Baxevanis, Sotirios P. Fortis

**Affiliations:** Cancer Immunology and Immunotherapy Center, Cancer Research Center, Saint Savas Cancer Hospital, 171 Alexandras Avenue, 11522 Athens, Greece; savstok@gmail.com (S.S.); costas.baxevanis@gmail.com (C.N.B.)

**Keywords:** HLA-alleles, HLA-A*02:01, HLA-A*24:02, prognosis, prostate cancer

## Abstract

Recently, we have shown that HLA-A*02:01 and HLA-A*24:02 in de novo metastatic prostate cancer (MPCa) have an important role in disease progression. Since de novo MPCa represents a small group among patients diagnosed with prostate cancer (PCa), it was obvious to try to extend the validity of our results to larger cohorts of PCa patients. Herein, we analyzed patients irrespective of their disease status at diagnosis to include, besides patients with MPCa, those with localized PCa (LPCa). Our goal was to specify the prognostic value of HLA-A*02:01 and HLA-A*24:02 for overall survival (OS) prospectively and for early biochemical recurrence (BCR) and castrate resistance (CR) as additional clinical endpoints in a prospective/retrospective manner, to improve clinical decisions for patients covering all stages of PCa. On univariate analysis, HLA-A alleles were significantly associated as prognostic biomarkers with early BCR (*p* = 0.028; HR = 1.822), OS (*p* = 0.013; HR = 1.547) and showed a trend for CR (*p* = 0.150; HR = 1.239). On multivariate analysis, HLA-A alleles proved to be independent prognosticators for early BCR (*p* = 0.017; HR = 2.008), CR (*p* = 0.005; HR = 1.615), and OS (*p* = 0.002; HR = 2.063). Kaplan–Meier analyses revealed that patients belonging to the HLA-A*02:01^+^HLA-A*24:02^−^ group progressed much faster to BCR and CR and had also shorter OS compared to HLA-A*24:02^+^ patients. Patients being HLA-A*02:01^−^HLA-A*24:02^−^ exhibited varying clinical outcomes, pointing to the presence of additional HLA-A alleles with potential prognostic value. Our data underline the HLA-A alleles as valuable prognostic biomarkers for PCa that may assist with the appropriate treatment and follow-up schedule based on the risk for disease progression to avoid over-diagnosis and over-treatment.

## 1. Introduction

Although prostate cancer (PCa)-related death incidence has substantially decreased by about 50% since the early nineties [1], it remains the second leading cause of cancer-related deaths in men [2]. PCa incidence and mortality vary widely among countries and ethnic populations [3]. The heterogeneous prognosis of PCa underlines the necessity of developing prognostic biomarkers that will pave the way towards personalized disease management [4]. In addition, PCa lacks or has limited expression of neoantigens as compared to other types of cancers, thus providing a serious obstacle for conventional immuno-therapeutic (immune checkpoint-based) regimens to achieve effective clinical responses [5]. Consequently, the discovery of prognostic biomarkers in this type of cancer is essential for the improvement of clinical efficacy through the design of appropriate treatment modalities. Although established clinicopathological parameters (e.g., prostate-specific antigen (PSA), Gleason score (GS), clinical T stage (cT), etc.) allow certain risk stratification, they are still not sufficient for the accurate prediction of clinical outcomes [6,7]. Among the plethora of parameters considered responsible for these worldwide and ethnic variations in PCa development and progression [8], the patient’s immune profile seems to be a key player.

The crucial role of the immune system in cancer is indisputable nowadays. The interaction between cancer cells and different immune cells is continuous, dynamic and greatly influences clinical outcomes [9]. Antigen presentation to T cells by the highly polymorphic HLA molecules is fundamental for eliciting adaptive antitumor responses [10]. HLA alleles have been related to the prevalence or outcome of several diseases, including autoimmunity and cancer [11].

The presence of certain HLA alleles has been shown to affect the survival outcome of cancer patients, and therefore, their role as prognostic biomarkers is gaining increasing attention [12,13,14]. Moreover, recent data have ascribed a predictive role to HLA alleles based on their involvement in determining patients’ clinical responses to immunotherapies via immune checkpoint blockade [15]. We have recently reported the favorable and unfavorable prognostic roles of the HLA-A*24:02 and HLA-A*02:01 alleles, respectively, in de novo metastatic PCa (MPCa) patients [16]. De novo MPCa represents only a small (currently about 5%) group among all PCa patients, which has dramatically decreased since the late eighties by about 60% due to the establishment of PSA screening [1]. The low incidence of de novo MPCa restricts the prognostic value of the HLA alleles, thus underscoring the need to analyze their prognostic potential in localized PCa (LPCa) patients, which constitute the vast majority of PCa cases.

Concerning the above, in the current study, we extended our investigation in PCa patients, irrespective of their disease status at diagnosis (i.e., LPCa or MPCa), to prospectively define the prognostic relevance of HLA-A*02:01 and HLA-A*24:02 to disease-related OS prediction as the ultimate prognostic endpoint at diagnosis, thus minimizing over-diagnosis. We also investigated whether these could additionally predict time to progression to intermediate endpoints during disease evolution. Our final goal is to provide another dynamic biomarker in the prognostic armamentarium of PCa to assist (i) in the optimal selection and timing of the required treatment modalities and (ii) in an appropriate follow-up interval frequency according to the risk of progression and death for each PCa patient. The successful approach of our goal could guide clinical decision-making to avoid overtreatments and improve the patients’ quality of life.

## 2. Results

### 2.1. Study Design and Patient Characteristics

Herein, we prospectively analyzed patients diagnosed with LPCa in addition to the 56 de novo MPCa patients from our recent study who, in the meantime, had a longer follow-up starting from December 2019 (latest clinical follow-up of our recent study [16] until September 2022 (last clinical follow-up of the present study), thus testing the prognostic value of these two alleles in an expanded cohort of PCa patients. A total of 204 PCa patients were enrolled, diagnosed between May 1998 and August 2019, of which 153 patients (75%), including 95 with LPCa and 58 with de novo MPCa (2 additional patients were enrolled in this group in the present study), were eligible because they met the criteria for fulfilling at least one clinical endpoint. These patients were of different clinical statuses at the time of enrolment, had complete clinical records, and were treated and clinically followed up at the Urology Clinic of Saint Savas Cancer Hospital. The remaining 51 patients had a short clinical follow-up from diagnosis and, therefore, could not be evaluated for any of the three clinical endpoints, i.e., biochemical recurrence (BCR), castrate resistance (CR), and overall survival (OS). Consequently, these patients were not included in the present study.

**Figure 1:**
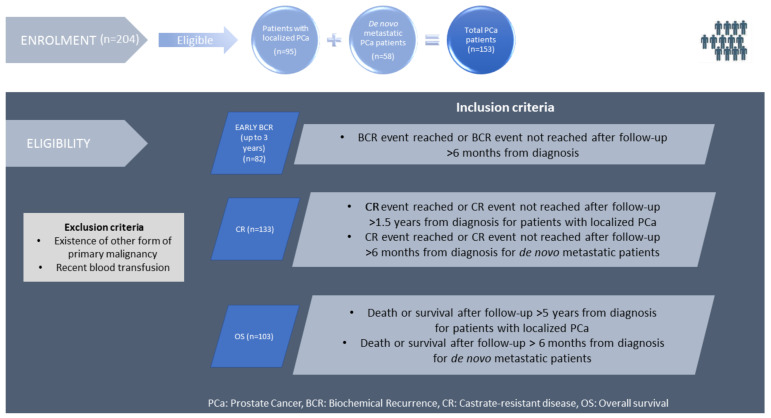
Enrolment and eligibility criteria of the study.

According to survival data reported by others [17] and the data from our patient cohort, evaluable LPCa patients for OS were those diagnosed at least 5 years before analysis unless death had occurred earlier (one patient in this cohort died at 3.5 years post-diagnosis). Patients diagnosed with LPCa in less than 5 years before analyses were excluded due to the extended time period needed to reach a death event. De novo MPCa patients were evaluable when diagnosed at least 6 months before analysis (providing sufficient time for recording potential death events) unless the death event was reached earlier. According to these limitations, from the total evaluable 153 patients, 103 patients were evaluable for OS, including 45 with LPCa and 58 with MPCa (Figure 1 and Supplementary Table S4). The detailed characteristics of these patients (prognostic clinicopathological characteristics, imaging data for metastatic burden evaluation, therapeutic approach selected by the treating urologist according to the standard of care for each patient, HLA typing of A locus, and years from diagnosis to event or censoring) are presented in Supplementary Table S1.

Early BCR, defined as occurring within 3 years from diagnosis and being predictive for shorter OS [18], could be investigated in patients diagnosed with LPCa. Finally, time to CR, already documented in our recent work in de novo MPCa as being significantly affected by different HLA-A alleles [16], was also evaluated in patients initially diagnosed with LPCa or MPCa. Again, based on published data [17] and observations from our cohort, LPCa patients evaluable for BCR and CR should have been diagnosed at least 6 months and 1.5 years, respectively, before being analyzed. De novo MPCa patients were evaluable for CR when diagnosed at least 6 months before analysis unless the event had occurred earlier. The eligibility criteria for each endpoint are summarized in Figure 1.

**Figure 2:**
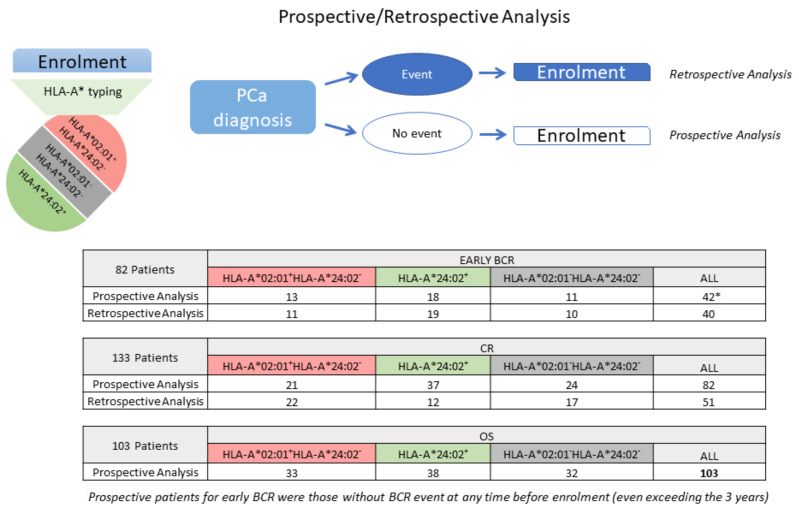
Flowchart of the study design and the patients’ enrolment.

The detailed characteristics (the same as those described for OS) of the PCa patients evaluated for early BCR (*n* = 82) or CR (*n* = 133) are presented in Supplementary Tables S2 and S3, respectively. The clinical characteristics of each cohort are summarized in Supplementary Table S4. As can be deduced from Supplementary Tables S1–S3, some patients were evaluable for more than one clinical endpoint. The number of patients analyzed prospectively or retrospectively for each endpoint is presented in Figure 2.

### 2.2. The Prognostic Significance of Specific HLA-A Alleles for OS

The median follow-up period from diagnosis for all 103 patients evaluable for OS was 5.56 years (range 0.46–22.27), which differed between LPCa (median 9.96 years; range 3.51–22.27) and MPCa (median 3.855 years; range 0.46–15.96). To investigate if different genotypes for HLA-A alleles, namely HLA-A*24:02^+^, HLA-A*02:01^+^HLA-A*24:02^−^, or negative for both alleles, were correlated with OS as previously documented for de novo MPCa patients [16], we initially conducted univariate and multivariate analyses (Table 1), including as covariates all the objective laboratory and imaging parameters considered to be prognostic for OS from the initial diagnosis [7,19], i.e., age, PSA, GS classified as ISUP grade groups [20], % of positive biopsies (>34%), clinical T stage (cT), presence of metastatic disease, and volume/burden of metastatic disease, if present. We also included as the primary therapy selected by the urologist according to the standard of care for each patient’s clinical and physical status (radical prostatectomy (RP), local radiation (PRTX), with or without androgen deprivation (ADT), and ADT alone) a parameter. Eighty-nine out of 103 patients, with no missing values, were included in the multivariate analysis. As presented in Table 1, the HLA-A allele genotype proved to be a significant prognosticator in the univariate analysis (*p* = 0.013). More importantly, in the multivariate analysis, HLA-A was found to be an independent OS prognostic biomarker at diagnosis for all PCa patients (LPCa and MPCa) (*p* = 0.002). Other independent prognostic factors were the Gleason score/ISUP (*p* = 0.005) and the volume of metastatic disease (*p* < 0.001).

**Figure 3:**
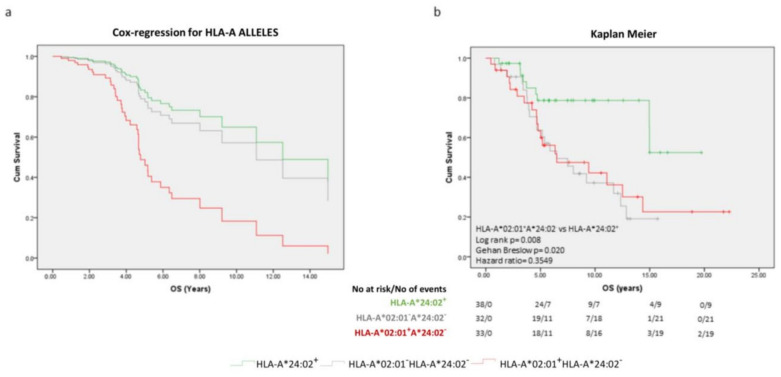
(**a**) Cox regression survival curves for model-predicted time to OS. (**b**) Kaplan–Meier curves illustrate time to OS in the indicated groups of patients. Statistical differences and hazard ratios among groups are also reported.

As shown in Figure 3a, from the Cox regression survival curves taking into account all the covariates, HLA-A*24:02 was found to be a good prognosticator, and HLA-A*02:01 (in the absence of HLA-A*24:02), the worst OS prognosticator at diagnosis, far worse than the simultaneous absence of the two alleles, which was closer to the favorable A*24:02^+^ group. Similar patterns for OS were also observed when patients (HLA-A*24:02^+^ vs. HLA-A*02:01^+^HLA-A*24:02^−^ vs. double negative) were stratified by volume of metastatic disease or ISUP (Supplementary Figure S1).

To obtain comparisons of the time and probability of death from the initial diagnosis, we applied a Kaplan–Meier analysis for the three different genotype groups without any other stratifications due to the small number of patients (Figure 3b). With a median OS of 11.08 years, this was estimated at 6.49 years and 6.48 years for the HLA-A*02:01^+^HLA-A*24:02^−^ and the HLA-A*02:01^−^HLA-A*24:02^−^ patients, respectively. The median OS survival could not be determined for the HLA-A*24:02^+^ patients. The three- and five-year probability of surviving was 97.4% and 78.7% for the HLA-A*24:02^+^ group, 90.6% and 63.8% for the double negative patients, and 80.9% and 63.4% for the A*02:01^+^HLA^−^A*24:02^−^ cohort, respectively.

In the univariate analysis, the HLA-A alleles were significantly associated as a prognostic biomarker with early BCR (*p* = 0.028). In the multivariate analysis, the HLA-A alleles, along with the % biopsy positivity, proved to be independent prognosticators for BCR (*p* = 0.017 and *p* = 0.002, respectively). In the univariate analysis for CR, the HLA-A alleles showed only a trend (*p* = 0.150) as prognosticators, whereas the multivariate analysis highlighted them as strong independent prognostic biomarkers (*p* = 0.005), along with GS/ISUP (*p* = 0.022), cT (*p* = 0.034) and metastatic burden/volume (*p* = 0.004) (Table 2).

**Figure 4:**
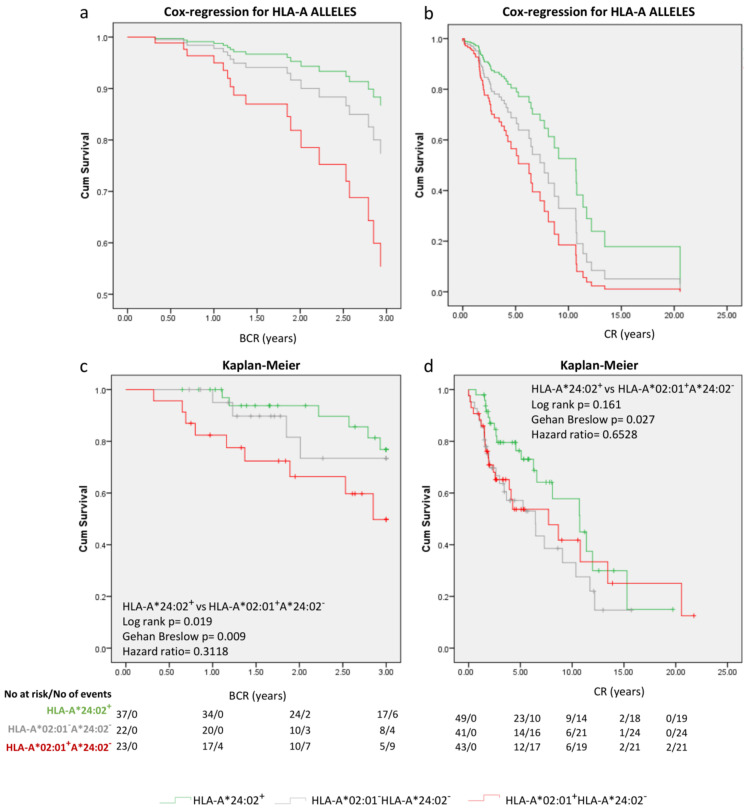
(**a**,**b**) Cox regression survival curves for model-predicted time to BCR and CR, respectively. The Kaplan–Meier curves illustrate time to BCR (**c**) and CR (**d**) in the indicated groups of patients. Statistical differences and hazard ratios among groups are also reposted.

As seen in the Cox regression survival curves (Figure 4a,b), patients with the HLA-A*24:02 genotype had the best clinical outcome in terms of early BCR and CR, whereas HLA-A*02:01^+^HLA-A*24:02^−^ showed poor clinical outcomes with the double negative cohort exhibiting intermediate clinical outcomes. Stratifications by significant covariates for both BCR (Supplementary Figure S2) and CR (Supplementary Figure S3) revealed similar patterns for prognosis for the 3 cohorts. Kaplan–Meier analyses (Figure 4c,d) for the three groups of HLA-A genotypes (without other stratifications) revealed that the probability for not having early BCR at 1, 2, and 3 years was 100%, 93.8% and 76.8%, respectively, for HLA-A*24:02^+^ LPCa, 95%, 81.6% and 73.4% for the double negative group and 82.4%, 66.3%, and 49.8% for the HLA-A*02:01^+^HLA-A*24:02^−^ patients, respectively. The median time to BCR could not be calculated. Similarly, the possibility of not becoming CR at 1, 5, and 10 years was 98%, 76.4%, and 57.8% for the HLA-A*24:02^+^ PCa group, 90.2%, 57.1%, and 33.1% for the double negative group and 90.7%, 53.7%, and 41.8% for the HLA-A*02:01^+^HLA-A*24:02^−^ patients, respectively. The median time to CR was 11.08 years for the whole cohort, 6.48 years for the double negative group, and 6.49 years for the HLA-A*02:01^+^HLA-A*24:02^−^ patients. For the favorable HLA-A*24:02^+^ group, the median time to CR could not be defined.

### 2.3. Focusing on HLA-A*02:01 and HLA-A*24:02 as Prognosticators at the Diagnosis of PCa

**Figure 5:**
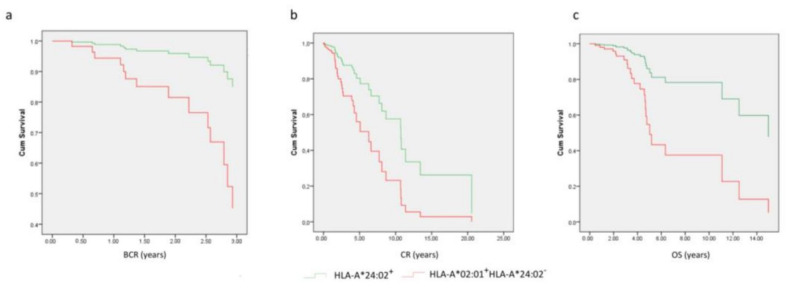
Cox regression survival curves for the model-predicted time to (**a**) BCR, (**b**) CR, and (**c**) OS.

We have so far defined two HLA-A sub-alleles, namely HLA-A*02:01 and HLA-A*24:02, with clearly opposite, direct, or indirect, effects on the clinical progression of PCa from diagnosis to early BCR (for LPCa patients treated with localized therapy), CR and, more importantly to death (OS). To have a closer look at the correlations of these alleles with the different examined endpoints, we applied univariate and multivariate analyses only in patients bearing these alleles (Table 3 and Figure 5). Patients genotyped positive for HLA-A*02:01 (but lacking HLA-A*24:02) were at higher risk for early BCR (*p* = 0.026) or death (*p* = 0.011), with only a trend for earlier CR (*p* = 0.165) compared to their HLA-A*24:02^+^ counterparts, as revealed in the univariate analysis. Although the number of patients without any missing values that were available for multivariate analyses was significantly reduced (*n* = 57 for early BCR, *n* = 86 for CR, and n= 64 for OS), again, the two alleles were proven strong independent prognostic factors for early BCR (*p* = 0.009), CR (*p* = 0.006) and OS (*p* = 0.002), along with % positive biopsies (*p* = 0.001) for early BCR, GS/ISUP (*p* = 0.001) and primary therapy *p* ≤ 0.001) for CR, and GS/ISUP (*p* = 0.019) and metastatic burden/volume (*p* < 0.001) for OS. It is obvious that when selecting RP, PRTX, or ADT as primary therapy, the time to CR is significantly affected since for each therapeutic modality, patients are exposed to ADT for shorter or longer periods from the initial diagnosis, and thus, ADT results in delayed or earlier development of resistance to androgen deprivation from the time of diagnosis.

It is important to re-emphasize here, that the relation of these two alleles with OS has been established by following patients prospectively, without any information regarding their HLA genotype at the enrolment.

## 3. Discussion

In the present study, we confirm and extend our recent data [16] by demonstrating the independent good or bad prognostic significance of specific HLA-A alleles, namely HLA-A*24:02 and HLA-A*02:01, respectively, for OS risk prediction at diagnosis, in a prospective cohort of localized or metastatic PCa patients initially diagnosed at any stage of the disease. We additionally show that these alleles constitute strong prognosticators for early BCR and CR in PCa patients retrospectively and prospectively analyzed based on whether or not they have reached the particular endpoint at the time of enrolment. Those findings confirm our previously published results in a de novo MPCa cohort [16], as well as in a cohort of LPCa, in which a correlation between HLA-A*02:01 expression and increased risk of disease recurrence after radical prostatectomy, independent of the clinicopathological characteristics, is reported [21]. Our varying data in patients lacking either the HLA-A*24:02 or the HLA-A*02:01 genotype or both, further indicate that the HLA-A locus-dependent prognostic significance at diagnosis may not be restricted only to these two alleles, but also other HLA-A alleles that are relatively under-represented in the Greek population may have a prognostic potential for worse or better clinical outcomes in PCa.

In our univariate and multivariate analyses for OS prediction, we used all of the clinicopathological characteristics from established prognostic models in a combined manner for LPCa and MPCa patients [7,19,22]. The same characteristics were also properly applied for CR and early BCR at diagnosis. All analyses demonstrated that HLA-A*24:02 and A*02:01 genotypes are independent predictors for PCa clinical outcomes at diagnosis.

It is impressive that in the prospective evaluation of OS, as well as in the combined prospective/retrospective analysis for both early BCR and CR, the favorable clinical outcome of the A*24:02^+^ cohorts compared to their HLA-A*02:01^+^A*24:02^−^ counterparts also remained after the stratification by other independent covariates. These included tumor burden as defined by the tumor volume for OS and CR, cT for CR, or % of positive biopsies for early BCR, and differentiation status, as described by the ISUP categorized Gleason Score for both OS and CR.

The independent prognostic potential of specific HLA-A alleles for OS for the de novo MPCa patients from our recent report [16] could be explained by the advanced nature of the disease at diagnosis, possibly as a result of a continuous immune-based selection for escape from immunosurveillance progressing to metastasis [23]. However, the fact that we have noticed the same prognostic pattern for HLA-A*02:01 and A*24:02 genotypes relative to disease progression to early BCR in patients diagnosed with localized disease proposes that these HLA-A alleles importantly and inherently contribute to PCa evolution independent of the disease status or treatments at diagnosis.

There are reports in the literature to show the prognostic role of specific HLA genotypes in several cancers, either for better or worse clinical outcomes differing among disease types [13,24,25,26,27,28,29,30]. For instance, in line with our recent data [16], HLA-A2 has been related to bad prognosis for ovarian [12], lung [14],, and prostate cancer [31], although this allele showed no prognostic relevance in breast cancer [32] and melanoma [13]. Intriguingly and in contrast with our data presented herein and earlier [16], the HLA-A*24 genotype has been included among the bad prognosticators for melanoma and lung cancer [13,29]. These discrepant observations indicate that the mechanisms underlying the involvement of certain HLA-A genotypes in cancer evolution differ among cancer types and are related to the natural history and complex biology of various malignancies. Consequently, they may indicate a predisposition for a certain cancer type, or their negative impact may be predominantly influenced by specific mutations and immune selection or by mechanisms leading to differential epigenetic modulation of allele expression. In PCa, HLA-A allele mutations detected so far are very rare [33,34], thus excluding the possibility of any contribution to the prognostic potential of HLA-A*02:01 and HLA-A*24:02. HLA-A*02:01 has been shown to restrict HLA-A-specific recognition of various tumor peptides by T lymphocytes [35] thus showing a prominent role in the design of therapeutic cancer vaccines and also chimeric antigen receptor T cell-based immunotherapies [36]. Consequently, one could expect a favorable prognostic role of HLA-A*02:01 in the PCa patient cohorts examined herein. To provide a possible explanation for this in the context of the immunoediting theory [37], we could propose an immune-driven pressure for the selection of tumor clones not efficiently recognized by HLA-A*02:01-restricted T cells, thus inhibiting the generation of robust antitumor immunity. To this end, in our previous reports, we demonstrated modest or no clinical responses in HLA-A*02:01^+^ PCa patients vaccinated with a modified HER-2/neu polypeptide, although these patients exhibited strong preexisting immunity to a variety of PCa-associated peptides [38,39].

No significant differences in the HLA-A*02:01 allele frequency have been observed among our PCa patients compared to the normal population. On the other hand, HLA-A*24:02-restricted CD8+ T cell antitumor immune responses have been detected in PCa [38,40], in many cases being much stronger than those restricted by HLA-A*02:01, making it unlikely that immune selection of tumor cell escape variants could be the predominant mechanism for HLA-A genotype-related disease prognosis. Thus, epigenetic regulation of these alleles is more plausible. For instance, it is quite interesting that HLA-A*24 is less prone to methylation than HLA-A*02 [41].

Interestingly, our varying data on the prognostic role of the HLA-A*02:01^−^HLA-A*24:02^−^ group(s), most possibly due to differences in HLA genotypes among the different cohorts, propose that other alleles or combinations of alleles or superfamilies may also define groups of patients at higher or lower risk for more aggressive disease than others. Apart from this, our data clearly show that the HLA-A*24:02 genotype is a very strong prognosticator for PCa, in contrast to HLA-A*02:01. However, we cannot exclude the possibility that other HLA-A genotypes may also predict better or worse clinical outcomes.

The hypothesis of epigenetic regulation as the predominant underlying mechanism for such differences among HLA-A alleles supports the possibility that superfamilies/supertypes bearing similar functional sequences at the protein level [42,43,44] may also contain similar DNA and/or mRNA sequences related to common epigenetic regulation. Such common functional sequences among members of the HLA-A superfamilies will determine common intermolecular interaction profiles with clustered functional properties. Under this view, preliminary analyses of the prognostic role of specific superfamilies (or superfamily member alleles) in our PCa patient cohort indicate that this may hold true.

The limitation of the current study is the relatively small number of PCa patients evaluated, either prospectively or retrospectively. However, it would be fair to emphasize that the prospective evaluation is a difficult task for rather indolent cancer types, requiring extended follow-up periods (more than 5–10 years, depending on the investigated endpoint). Nevertheless, our prospective cohorts comprising all patients analyzed for OS and almost half of those analyzed for early BCR and/or CR, along with the similar findings among the different clinical endpoints examined, partly counteract the limited number of patients, and strongly support the significant prognostic value of HLA-A*02:01 and HLA-A*24:02. We believe that our data will be strengthened via increasing the number of evaluable patients for the different endpoints not only by longer clinical follow-up of the total PCa population enrolled in this study but also by incorporating underrepresented alleles and their corresponding superfamilies in our analyses.

## 4. Materials and Methods

A retrospective review of the medical records of 204 patients (146 with LPCa and 58 de novo metastatic PCa) from the “Saint Savas Cancer Hospital” in Greece was performed between March 2017 and April 2020. Written informed consent was obtained from all of the enrolled patients. The study and the informed consent form were approved by the Hospital IRB (IRB-ID6777/14-06-2017) and the Ethical Committee of the University of Athens (IRB-ID1516015872/03-02-2016). Patients enrolled in this combined prospective/retrospective study had already received or were planned to receive standard medical treatment upon diagnosis and had complete medical records, including baseline disease characteristics, received treatments and clinical follow-up before and after the enrolment. The eligible patients with LPCa (*n* = 95) at diagnosis had either developed early BCR or had a clinical follow-up >6 months from diagnosis without any BCR event. For CR, an event had to be reached. Otherwise, a follow-up of 1.5 years from diagnosis was required. Follow-up >5 years from diagnosis or death having occurred at any earlier time point after enrolment was also required for the eligibility of LPCa patients for OS evaluation. For de novo MPCa patients to be eligible (*n* = 58), a minimum follow-up of 6 months from diagnosis was required for CR and OS, unless earlier CR had occurred (Figure 1). Patients with other primary malignancies or with a recent blood transfusion were excluded. Blood for HLA typing was collected at the time of enrolment. The endpoints of the study were (i) time to early BCR from diagnosis for patients diagnosed with LPCa, (ii) CR from diagnosis, and (iii) OS from diagnosis. Early BCR was defined as that occurred in the first 3 years after local treatment [18]. BCR was defined as two consecutive PSA values ≥ 0.2 ng/mL after RP [45] or PSA > nadir + 2 ng/mL after PRTX [46]. Clinical evaluation was assessed according to Response Evaluation Criteria In Solid Tumors (RECIST) (Version 1.1) [47]. The clinicopathological characteristics of PCa patients eligible for early BCR, CR, or OS at diagnosis are presented in Supplementary Table S4. The ISUP 2014/WHO 2016 grading system was used for our analyses [48]. The percentages of positive cores in the prostate needle biopsy (>34%) [49] and metastatic PCa burden (i.e., low or high volume) among other baseline clinicopathological characteristics were evaluated. High volume was defined as the presence of visceral metastases or 4 or more bone metastases with >1 bone lesion beyond the pelvis or the axis [50]. The individual clinicopathological characteristics, primary therapy, clinical outcome, and HLA-A genotype for patients evaluated for OS, early BCR, and CR are separately shown in Supplementary Tables S1–S3, respectively, despite some overlap in patients examined for more than one endpoint.

### 4.1. HLA Typing

HLA-A class I antigen genotyping was performed using next-generation sequencing for the A-locus (ONE LAMBDA Inc, ThermoFisher Scientific, Waltham, MA, USA).

### 4.2. Statistical Analysis

The IBM SPSS version 24.0.0.1 software was used for all statistical analyses. Cumulative survival probabilities testing using the Kaplan–Meier analysis with 95% confidence intervals (95%-CIs) was performed to evaluate the possible association between HLA expression and clinical outcome. The survival curves were calculated and compared using the log-rank test (Mantel–Cox) and the Gehan–Breslow–Wilcoxon test, mentioning the Hazard Ratio (HR; log rank). Statistical differences were considered significant for *p* values < 0.05. Values between 0.1–0.05 were considered a trend. Univariate and multivariate survival analyses (Cox regression) were also conducted. For the multivariate analysis, the forward stepwise method with a threshold of 0.05 as an entry point was used.

## 5. Conclusions

Specific HLA-A alleles, including, but not restricted to, those described here, i.e., HLA-A*02:01 and HLA-A*24:02, as independent prognostic biomarkers, may change therapeutic and clinical follow-up time scheduling, thus significantly contributing to overcome over-diagnosis and over-treatment of PCa patients. For instance, in accordance with the newly suggested guidelines [51] and according to our data from this study, patients carrying the HLA-A*24:02 genotype and experiencing BCR after local therapy may start ADT after metastatic disease diagnosis, thus avoiding for a long time the significant side effects of anti-androgens, and consequently enjoying an improved quality of life for longer periods. On the contrary, PCa patients with more aggressive disease, as those HLA-A*02:01 genotyped, should immediately start appropriate treatment schemes to delay metastases (including ADT, second-line anti-androgens, and/or chemotherapy) according to other risk-indicative clinicopathological characteristics.

Apart from HLA-A alleles being used as prognosticators, the underlying mechanisms controlling their expression should be investigated as this could lead to therapeutic interventions aiming at restoring effective HLA-A allele expression.

## Figures and Tables

**Table 1:** Univariate and multivariate analysis for the association of risk factors with OS.

Univariate	OS		
	HR	95.0% CI for HR (Range)	*p*-Value
HLA-A allele	1.547	1.098–2.181	0.013
Age	1.96	1.323–2.920	0.001
PSA	1.434	1.149–1.790	0.001
ISUP Grade group	1.686	1.320–2.153	<0.0001
% Positive biopsy	2.577	1.091–6.083	0.031
Metastasis	6.634	3.381–13.017	<0.0001
cT	1.886	1.451–2.452	<0.0001
Volume	3.269	2.242–4.766	<0.0001
Primary Therapy	3.119	1.973–4.931	<0.0001
**Multivariate**	**OS**		
	**HR**	**95.0% CI for HR** **(Range)**	***p*** **-Value**
Model before Stepwise Selection			
HLA-A allele	2.039	1.268–3.279	0.003
Age	1.127	0.671–1.895	0.652
PSA	1.009	0.745–1.367	0.952
ISUP Grade group	1.377	1.009–1.881	0.044
% Positive biopsy	1.506	0.440–5.155	0.514
Metastasis	0.180	0.010–3.336	0.250
cT	1.842	0.735–4.613	0.192
Volume	2.614	1.254–5.449	0.010
Primary Therapy	1.535	0.533–4.420	0.427
Model after Stepwise Selection			
HLA-A allele	2.063	1.300–3.275	0.002
ISUP Grade group	1.516	1.136–2.024	0.005
Volume	3.071	1.900–4.966	<0.0001

OS: overall survival; CI: confidence interval; PSA: prostate-specific antigen; ISUP: International Society of Urological Pathology; HR: hazard ratio. All categorical covariates were transformed into numeric codes as follows: HLA-A allele: HLA-A*24:02^+^, 1; HLA-A*02:01^−^HLA-A*24:02^−^, 2; and HLA-A*02:01^+^HLA-A*24:02^−^, 3; Age: <65, 1; 65–75, 2; and >75, 3; PSA: ≤10, 1; >10 ≤ 20, 2; >20 ≤ 100, 3; >100 ≤ 500, 4; >500, 5; ISUP grade group: ISUP 1 (GS ≤ 6), 1; ISUP 2 (GS 3 + 4), 2; ISUP 3 (GS 4 + 3), 3; ISUP 4 (GS = 8), 4; and ISUP 5 (GS ≥ 9), 5; % positive biopsy: percent of positive cores in the prostate needle biopsy ≤34%, 1; and percent of positive cores in the prostate needle biopsy >34%, 2; Metastasis: absent, 1 and present, 2; cT: T1a-T1c, 1; T2a-T2c, 2; T3a, 3; T3b, 4; and T4, 5; Volume: no metastasis, 0; low, 1; and high (visceral metastases and/or 4 or more bone metastases), 2; and Primary Therapy: Radical Prostatectomy, 1; Primary Radiotherapy, 2; and Androgen deprivation therapy, 3.

**Table 2:** Univariate analysis and multivariate analysis of the risk factors for early BCR and CR.

Univariate	Early BCR			CR		
	HR	95.0% CI for HR (Range)	*p*-Value	HR	95.0% CI for HR (Range)	*p*-Value
HLA-A allele	1.822	1.068–3.109	0.028	1.239	0.925–1.660	0.150
Age	0.403	0.175–0.929	0.033	1.657	1.173–2.342	0.004
PSA	0.871	0.327–2.323	0.783	3.033	1.782–5.162	<0.0001
ISUP Grade group	1.310	0.946–1.815	0.104	1.623	1.326–1.988	<0.0001
% Positive biopsy	3.403	1.225–9.450	0.019	1.990	1.054–3.758	0.034
Metastasis	n/a	n/a	n/a	8.206	4.696–14.337	<0.0001
cT	1.679	1.054–2.677	0.029	2.056	1.637–2.581	<0.0001
Volume	n/a	n/a	n/a	4.031	2.846–5.709	<0.0001
Primary Therapy	0.125	0.017–0.941	0.043	2.964	2.097–4.190	<0.0001
**Multivariate**	**Early BCR**			**CR**		
	**HR**	**95.0% CI for HR (Range)**	***p*** **-Value**	**HR**	**95.0% CI for HR (Range)**	***p*** **-Value**
Model before Stepwise Selection						
HLA-A allele	2.122	1.135–3.968	0.019	1.625	1.131–2.333	0.009
Age	0.564	0.204–1.561	0.270	1.108	0.742–1.655	0.617
PSA	0.887	0.277–2.837	0.840	1.009	0.488–2.084	0.981
ISUP Grade group	0.813	0.518–1.274	0.366	1.291	1.011–1.647	0.040
% Positive biopsy	7.308	1.894–28.196	0.004	1.009	0.494–2.061	0.981
Metastasis	n/a	n/a	n/a	0.584	0.073–4.662	0.612
cT	1.103	0.576–2.114	0.767	1.538	0.873–2.709	0.136
Volume	n/a	n/a	n/a	2.136	1.063–4.293	0.033
Primary Therapy	0.124	0.013–1.164	0.068	1.336	0.663–2.691	0.418
Model after Stepwise Selection						
HLA-A allele	2.008	1.135–3.551	0.017	1.615	1.158–2.251	0.005
Positive biopsy	6.045	1.946–18.780	0.002	-	-	-
ISUP Grade group	-	-	-	1.313	1.039–1.658	0.022
cT	-	-	-	1.482	1.029–2.133	0.034
Volume	-	-	-	2.153	1.279–3.623	0.004

BCR: Biochemical Recurrence; CR: Castrate Resistance; CI: confidence interval; PSA: prostate-specific antigen; ISUP: International Society of Urological Pathology; HR: hazard ratio; n/a: not applicable. All categorical covariates were transformed into numeric codes as follows: HLA-A allele: HLA-A*24:02^+^, 1; HLA-A*02:01^−^HLA-A*24:02^−^, 2; and HLA-A*02:01^+^HLA-A*24:02^−^, 3; Age: <65, 1; 65–75, 2; and >75, 3; PSA: ≤20, 1; >20, 2; ISUP grade group: ISUP 1 (GS ≤ 6), 1; ISUP 2 (GS 3 + 4), 2; ISUP 3 (GS 4 + 3), 3; ISUP 4 (GS = 8), 4; and ISUP 5 (GS ≥ 9), 5; % positive biopsy: percent of positive cores in the prostate needle biopsy ≤34%, 1; and percent of positive cores in the prostate needle biopsy >34%, 2; Metastasis: absent, 1 and present, 2; cT: T1a-T1c, 1; T2a-T2c, 2; T3a, 3; T3b, 4; and T4, 5; Volume: no metastasis, 0; low, 1; and high (visceral metastases and/or 4 or more bone metastases), 2; and Primary Therapy: Radical Prostatectomy, 1; Primary Radiotherapy, 2; and Androgen deprivation therapy, 3.

**Table 3:** Univariate analysis and multivariate analysis of the risk factors for early BCR, CR, and OS.

Univariate	Early BCR			CR			OS		
	HR	95.0% CI for HR (Range)	*p*-Value	HR	95.0% CI for Exp(B) (Range)	*p*-Value	HR	95.0% CI for HR (Range)	*p*-Value
HLA-A allele	1.806	1.073–3.039	0.026	1.247	0.913–1.703	0.165	1.672	1.124–2.487	0.011
Age	0.510	0.201–1.292	0.156	1.685	1.067–2.660	0.025	1.960	1.126–3.306	0.012
PSA	0.936	0.325–2.699	0.903	2.731	1.427–5.223	0.002	1.353	1.003–1.826	0.048
ISUP Grade group	1.457	0.987–2.152	0.058	1.850	1.406–2.433	<0.0001	1.771	1.268–2.475	0.001
% Positive biopsy	3.883	1.234–12.217	0.020	1.831	0.889–3.770	0.101	1.896	0.767–4.690	0.166
Metastasis	n/a	n/a	n/a	7.694	3.860–15.335	<0.0001	6.578	2.792–15.501	<0.0001
cT	1.781	1.045–3.035	0.034	2.058	1.558–2.719	<0.0001	2.015	1.419–2.864	<0.0001
Volume	n/a	n/a	n/a	3.952	2.542–6.143	<0.0001	3.455	2.097–5.690	<0.0001
Primary Therapy	0.173	0.023–1.320	0.091	2.977	1.934–4.582	<0.0001	2.856	1.642–4.969	<0.0001
**Multivariate**	**Early BCR**			**CR**			**OS**		
	**HR**	**95.0% CI for HR (Range)**	***p*** **-Value**	**HR**	**95.0% CI for Exp(B) (Range)**	***p*** **-Value**	**HR**	**95.0% CI for HR (Range)**	***p*** **-Value**
Model before Stepwise Selection									
HLA-A allele	2.393	1.234–4.639	0.010	0.354	0.161–0.77	0.010	0.253	0.097–0.655	0.005
Age	0.916	0.300–2.796	0.877	1.142	0.692–1.885	0.604	0.956	0.491–1.86	0.894
PSA	0.664	0.183–2.406	0.533	1.134	0.474–2.714	0.778	0.934	0.612–1.425	0.75
ISUP Grade group	0.890	0.528–1.500	0.661	1.565	1.124–2.178	0.008	1.469	0.952–2.266	0.082
% Positive biopsy	10.040	1.895–53.181	0.007	1.132	0.499–2.569	0.768	1.52	0.426–5.421	0.519
Metastasis	n/a	n/a	n/a	0.209	0.010–4.424	0.315	0.154	0.005–4.506	0.278
cT	1.145	0.562–2.334	0.710	1.528	0.753–3.100	0.240	1.502	0.548–4.112	0.429
Volume	n/a	n/a	n/a	2.002	0.831–4.825	0.122	5.038	1.61315.734	0.005
Primary Therapy	0.297	0.028–3.163	0.314	2.219	0.810–6.083	0.121	1.57	0.486–5.075	0.451
Model after Stepwise Selection									
HLA-A allele	2.205	1.217–3.996	0.009	0.377	0.187–0.761	0.006	0.25	0.102–0.609	0.002
% Positive biopsy	8.605	2.191–33.795	0.002	-	-	-	-	-	-
Primary Therapy	-	-	-	2.795	1.765–4.425	<0.0001	-		-
ISUP Grade group	-	-	-	1.676	1.221–2.301	0.001	1.552	1.075–2.242	0.019
Volume	-	-	-	-	-	-	4.211	2.082–8.518	<0.0001

BCR: Biochemical recurrence; CR: Castrate Resistance; OS: overall survival; CI: confidence interval; PSA: prostate-specific antigen; ISUP: International Society of Urological Pathology; HR: hazard ratio; n/a: not applicable. All categorical covariates were transformed into numeric codes as follows: HLA-A allele: HLA-A*24:02^+^, 1; and HLA-A*02:01^+^HLA-A*24:02^−^, 2; Age: <65, 1; 65–75, 2; and >75, 3; PSA: ≤20, 1; and >20, 2; ISUP grade group: ISUP 1 (GS ≤ 6), 1; ISUP 2 (GS 3 + 4), 2; ISUP 3 (GS 4 + 3), 3; ISUP 4 (GS = 8), 4; and ISUP 5 (GS ≥ 9), 5; % positive biopsy: percent of positive cores in the prostate needle biopsy ≤34%, 1; and percent of positive cores in the prostate needle biopsy >34%, 2; Metastasis: absent, 1 and present, 2; cT: T1a-T1c, 1; T2a-T2c, 2; T3a, 3; T3b, 4; and T4, 5; Volume: no metastasis, 0; low, 1; and high (visceral metastases and/or 4 or more bone metastases), 2; and Primary Therapy: Radical Prostatectomy, 1; Primary Radiotherapy, 2; and Androgen deprivation therapy, 3.

## Data Availability

The data presented in this study are available upon reasonable request.

## Supplementary Materials

The following are available online at https://www.mdpi.com/article/10.3390/ijms241914454/s1.
