# Supplementary material for: The Prognostic Significance of Selected HLA Alleles on Prostate Cancer Outcome
Source: Int J Mol Sci. 2023 Sep 22;24(19):14454. doi: 10.3390/ijms241914454 (PMC10572221; doi:10.3390/ijms241914454)
Supplement: Supplementary file 1 [file ijms-24-14454-s001.zip › ijms-2504801-supplementary.pptx]

## Slide 1
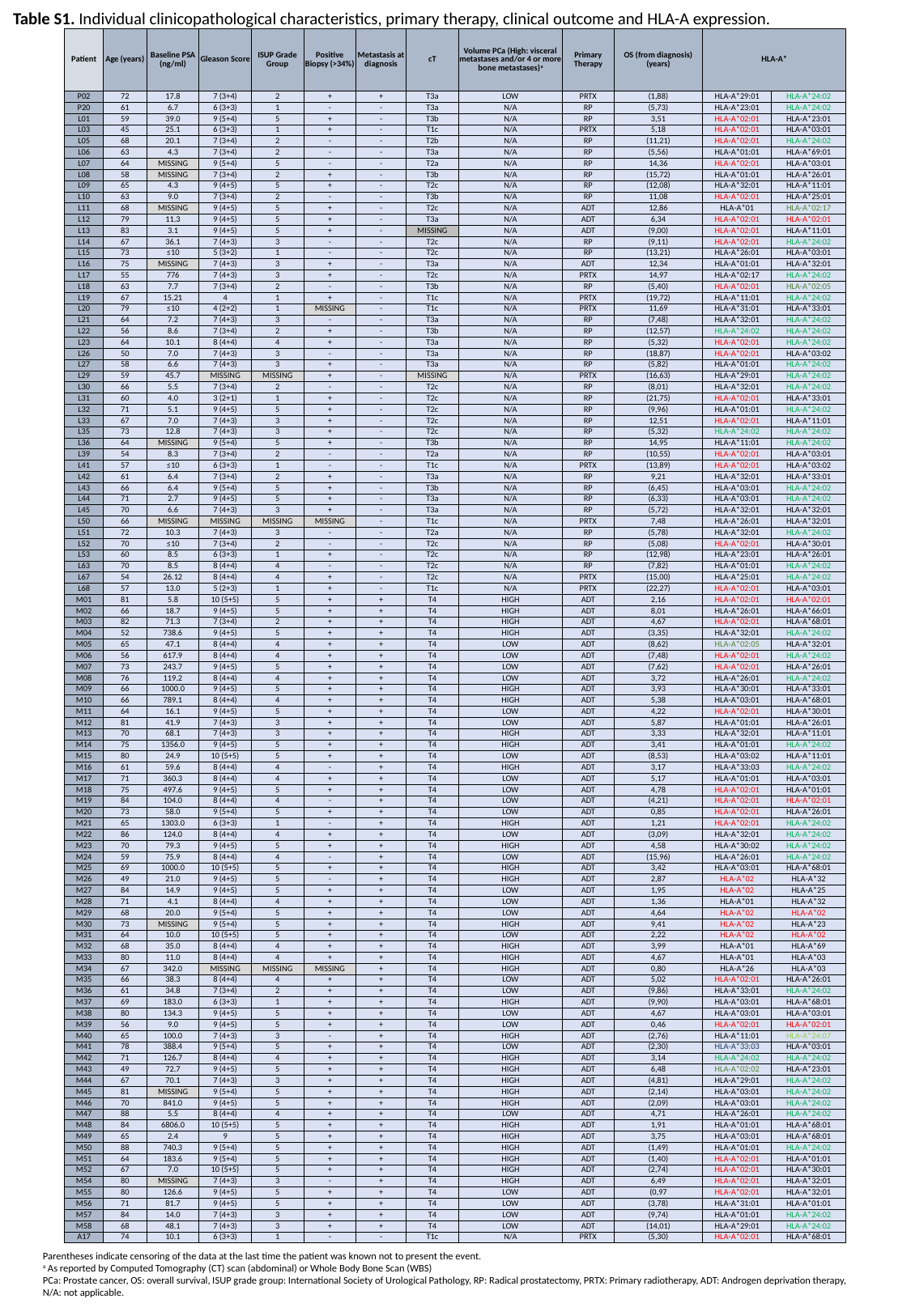

Table S1. Individual clinicopathological characteristics, primary therapy, clinical outcome and HLA-A expression.
| Patient | Age (years) | Baseline PSA (ng/ml) | Gleason Score | ISUP Grade Group | Positive Biopsy (>34%) | Metastasis at diagnosis | cT | Volume PCa (High: visceral metastases and/or 4 or more bone metastases)a | Primary Therapy | OS (from diagnosis) (years) | HLA-A\* | |
| --- | --- | --- | --- | --- | --- | --- | --- | --- | --- | --- | --- | --- |
| P02 | 72 | 17.8 | 7 (3+4) | 2 | + | + | T3a | LOW | PRTX | (1,88) | HLA-A\*29:01 | HLA-A\*24:02 |
| P20 | 61 | 6.7 | 6 (3+3) | 1 | - | - | T3a | N/A | RP | (5,73) | HLA-A\*23:01 | HLA-A\*24:02 |
| L01 | 59 | 39.0 | 9 (5+4) | 5 | + | - | T3b | N/A | RP | 3,51 | HLA-A\*02:01 | HLA-A\*23:01 |
| L03 | 45 | 25.1 | 6 (3+3) | 1 | + | - | T1c | N/A | PRTX | 5,18 | HLA-A\*02:01 | HLA-A\*03:01 |
| L05 | 68 | 20.1 | 7 (3+4) | 2 | - | - | T2b | N/A | RP | (11,21) | HLA-A\*02:01 | HLA-A\*24:02 |
| L06 | 63 | 4.3 | 7 (3+4) | 2 | - | - | T3a | N/A | RP | (5,56) | HLA-A\*01:01 | HLA-A\*69:01 |
| L07 | 64 | MISSING | 9 (5+4) | 5 | - | - | T2a | N/A | RP | 14,36 | HLA-A\*02:01 | HLA-A\*03:01 |
| L08 | 58 | MISSING | 7 (3+4) | 2 | + | - | T3b | N/A | RP | (15,72) | HLA-A\*01:01 | HLA-A\*26:01 |
| L09 | 65 | 4.3 | 9 (4+5) | 5 | + | - | T2c | N/A | RP | (12,08) | HLA-A\*32:01 | HLA-A\*11:01 |
| L10 | 63 | 9.0 | 7 (3+4) | 2 | - | - | T3b | N/A | RP | 11,08 | HLA-A\*02:01 | HLA-A\*25:01 |
| L11 | 68 | MISSING | 9 (4+5) | 5 | + | - | T2c | N/A | ADT | 12,86 | HLA-A\*01 | HLA-A\*02:17 |
| L12 | 79 | 11.3 | 9 (4+5) | 5 | + | - | T3a | N/A | ADT | 6,34 | HLA-A\*02:01 | HLA-A\*02:01 |
| L13 | 83 | 3.1 | 9 (4+5) | 5 | + | - | MISSING | N/A | ADT | (9,00) | HLA-A\*02:01 | HLA-A\*11:01 |
| L14 | 67 | 36.1 | 7 (4+3) | 3 | - | - | T2c | N/A | RP | (9,11) | HLA-A\*02:01 | HLA-A\*24:02 |
| L15 | 73 | ≤10 | 5 (3+2) | 1 | - | - | T2c | N/A | RP | (13,21) | HLA-A\*26:01 | HLA-A\*03:01 |
| L16 | 75 | MISSING | 7 (4+3) | 3 | + | - | T3a | N/A | ADT | 12,34 | HLA-A\*01:01 | HLA-A\*32:01 |
| L17 | 55 | 776 | 7 (4+3) | 3 | + | - | T2c | N/A | PRTX | 14,97 | HLA-A\*02:17 | HLA-A\*24:02 |
| L18 | 63 | 7.7 | 7 (3+4) | 2 | - | - | T3b | N/A | RP | (5,40) | HLA-A\*02:01 | HLA-A\*02:05 |
| L19 | 67 | 15.21 | 4 | 1 | + | - | T1c | N/A | PRTX | (19,72) | HLA-A\*11:01 | HLA-A\*24:02 |
| L20 | 79 | ≤10 | 4 (2+2) | 1 | MISSING | - | T1c | N/A | PRTX | 11,69 | HLA-A\*31:01 | HLA-A\*33:01 |
| L21 | 64 | 7.2 | 7 (4+3) | 3 | - | - | T3a | N/A | RP | (7,48) | HLA-A\*32:01 | HLA-A\*24:02 |
| L22 | 56 | 8.6 | 7 (3+4) | 2 | + | - | T3b | N/A | RP | (12,57) | HLA-A\*24:02 | HLA-A\*24:02 |
| L23 | 64 | 10.1 | 8 (4+4) | 4 | + | - | T3a | N/A | RP | (5,32) | HLA-A\*02:01 | HLA-A\*24:02 |
| L26 | 50 | 7.0 | 7 (4+3) | 3 | - | - | T3a | N/A | RP | (18,87) | HLA-A\*02:01 | HLA-A\*03:02 |
| L27 | 58 | 6.6 | 7 (4+3) | 3 | + | - | T3a | N/A | RP | (5,82) | HLA-A\*01:01 | HLA-A\*24:02 |
| L29 | 59 | 45.7 | MISSING | MISSING | + | - | MISSING | N/A | PRTX | (16,63) | HLA-A\*29:01 | HLA-A\*24:02 |
| L30 | 66 | 5.5 | 7 (3+4) | 2 | - | - | T2c | N/A | RP | (8,01) | HLA-A\*32:01 | HLA-A\*24:02 |
| L31 | 60 | 4.0 | 3 (2+1) | 1 | + | - | T2c | N/A | RP | (21,75) | HLA-A\*02:01 | HLA-A\*33:01 |
| L32 | 71 | 5.1 | 9 (4+5) | 5 | + | - | T2c | N/A | RP | (9,96) | HLA-A\*01:01 | HLA-A\*24:02 |
| L33 | 67 | 7.0 | 7 (4+3) | 3 | + | - | T2c | N/A | RP | 12,51 | HLA-A\*02:01 | HLA-A\*11:01 |
| L35 | 73 | 12.8 | 7 (4+3) | 3 | + | - | T2c | N/A | RP | (5,32) | HLA-A\*24:02 | HLA-A\*24:02 |
| L36 | 64 | MISSING | 9 (5+4) | 5 | + | - | T3b | N/A | RP | 14,95 | HLA-A\*11:01 | HLA-A\*24:02 |
| L39 | 54 | 8.3 | 7 (3+4) | 2 | - | - | T2a | N/A | RP | (10,55) | HLA-A\*02:01 | HLA-A\*03:01 |
| L41 | 57 | ≤10 | 6 (3+3) | 1 | - | - | T1c | N/A | PRTX | (13,89) | HLA-A\*02:01 | HLA-A\*03:02 |
| L42 | 61 | 6.4 | 7 (3+4) | 2 | + | - | T3a | N/A | RP | 9,21 | HLA-A\*32:01 | HLA-A\*33:01 |
| L43 | 66 | 6.4 | 9 (5+4) | 5 | + | - | T3b | N/A | RP | (6,45) | HLA-A\*03:01 | HLA-A\*24:02 |
| L44 | 71 | 2.7 | 9 (4+5) | 5 | + | - | T3a | N/A | RP | (6,33) | HLA-A\*03:01 | HLA-A\*24:02 |
| L45 | 70 | 6.6 | 7 (4+3) | 3 | + | - | T3a | N/A | RP | (5,72) | HLA-A\*32:01 | HLA-A\*32:01 |
| L50 | 66 | MISSING | MISSING | MISSING | MISSING | - | T1c | N/A | PRTX | 7,48 | HLA-A\*26:01 | HLA-A\*32:01 |
| L51 | 72 | 10.3 | 7 (4+3) | 3 | - | - | T2a | N/A | RP | (5,78) | HLA-A\*32:01 | HLA-A\*24:02 |
| L52 | 70 | ≤10 | 7 (3+4) | 2 | - | - | T2c | N/A | RP | (5,08) | HLA-A\*02:01 | HLA-A\*30:01 |
| L53 | 60 | 8.5 | 6 (3+3) | 1 | + | - | T2c | N/A | RP | (12,98) | HLA-A\*23:01 | HLA-A\*26:01 |
| L63 | 70 | 8.5 | 8 (4+4) | 4 | - | - | T2c | N/A | RP | (7,82) | HLA-A\*01:01 | HLA-A\*24:02 |
| L67 | 54 | 26.12 | 8 (4+4) | 4 | + | - | T2c | N/A | PRTX | (15,00) | HLA-A\*25:01 | HLA-A\*24:02 |
| L68 | 57 | 13.0 | 5 (2+3) | 1 | + | - | T1c | N/A | PRTX | (22,27) | HLA-A\*02:01 | HLA-A\*03:01 |
| M01 | 81 | 5.8 | 10 (5+5) | 5 | + | + | T4 | HIGH | ADT | 2,16 | HLA-A\*02:01 | HLA-A\*02:01 |
| M02 | 66 | 18.7 | 9 (4+5) | 5 | + | + | T4 | HIGH | ADT | 8,01 | HLA-A\*26:01 | HLA-A\*66:01 |
| M03 | 82 | 71.3 | 7 (3+4) | 2 | + | + | T4 | HIGH | ADT | 4,67 | HLA-A\*02:01 | HLA-A\*68:01 |
| M04 | 52 | 738.6 | 9 (4+5) | 5 | + | + | T4 | HIGH | ADT | (3,35) | HLA-A\*32:01 | HLA-A\*24:02 |
| M05 | 65 | 47.1 | 8 (4+4) | 4 | + | + | T4 | LOW | ADT | (8,62) | HLA-A\*02:05 | HLA-A\*32:01 |
| M06 | 56 | 617.9 | 8 (4+4) | 4 | + | + | T4 | LOW | ADT | (7,48) | HLA-A\*02:01 | HLA-A\*24:02 |
| M07 | 73 | 243.7 | 9 (4+5) | 5 | + | + | T4 | LOW | ADT | (7,62) | HLA-A\*02:01 | HLA-A\*26:01 |
| M08 | 76 | 119.2 | 8 (4+4) | 4 | + | + | T4 | LOW | ADT | 3,72 | HLA-A\*26:01 | HLA-A\*24:02 |
| M09 | 66 | 1000.0 | 9 (4+5) | 5 | + | + | T4 | HIGH | ADT | 3,93 | HLA-A\*30:01 | HLA-A\*33:01 |
| M10 | 66 | 789.1 | 8 (4+4) | 4 | + | + | T4 | HIGH | ADT | 5,38 | HLA-A\*03:01 | HLA-A\*68:01 |
| M11 | 64 | 16.1 | 9 (4+5) | 5 | + | + | T4 | LOW | ADT | 4,22 | HLA-A\*02:01 | HLA-A\*30:01 |
| M12 | 81 | 41.9 | 7 (4+3) | 3 | + | + | T4 | LOW | ADT | 5,87 | HLA-A\*01:01 | HLA-A\*26:01 |
| M13 | 70 | 68.1 | 7 (4+3) | 3 | + | + | T4 | HIGH | ADT | 3,33 | HLA-A\*32:01 | HLA-A\*11:01 |
| M14 | 75 | 1356.0 | 9 (4+5) | 5 | + | + | T4 | HIGH | ADT | 3,41 | HLA-A\*01:01 | HLA-A\*24:02 |
| M15 | 80 | 24.9 | 10 (5+5) | 5 | + | + | T4 | LOW | ADT | (8,53) | HLA-A\*03:02 | HLA-A\*11:01 |
| M16 | 61 | 59.6 | 8 (4+4) | 4 | - | + | T4 | HIGH | ADT | 3,17 | HLA-A\*33:03 | HLA-A\*24:02 |
| M17 | 71 | 360.3 | 8 (4+4) | 4 | + | + | T4 | LOW | ADT | 5,17 | HLA-A\*01:01 | HLA-A\*03:01 |
| M18 | 75 | 497.6 | 9 (4+5) | 5 | + | + | T4 | LOW | ADT | 4,78 | HLA-A\*02:01 | HLA-A\*01:01 |
| M19 | 84 | 104.0 | 8 (4+4) | 4 | - | + | T4 | LOW | ADT | (4,21) | HLA-A\*02:01 | HLA-A\*02:01 |
| M20 | 73 | 58.0 | 9 (5+4) | 5 | + | + | T4 | LOW | ADT | 0,85 | HLA-A\*02:01 | HLA-A\*26:01 |
| M21 | 65 | 1303.0 | 6 (3+3) | 1 | - | + | T4 | HIGH | ADT | 1,21 | HLA-A\*02:01 | HLA-A\*24:02 |
| M22 | 86 | 124.0 | 8 (4+4) | 4 | + | + | T4 | LOW | ADT | (3,09) | HLA-A\*32:01 | HLA-A\*24:02 |
| M23 | 70 | 79.3 | 9 (4+5) | 5 | + | + | T4 | HIGH | ADT | 4,58 | HLA-A\*30:02 | HLA-A\*24:02 |
| M24 | 59 | 75.9 | 8 (4+4) | 4 | - | + | T4 | LOW | ADT | (15,96) | HLA-A\*26:01 | HLA-A\*24:02 |
| M25 | 69 | 1000.0 | 10 (5+5) | 5 | + | + | T4 | HIGH | ADT | 3,42 | HLA-A\*03:01 | HLA-A\*68:01 |
| M26 | 49 | 21.0 | 9 (4+5) | 5 | - | + | T4 | HIGH | ADT | 2,87 | HLA-A\*02 | HLA-A\*32 |
| M27 | 84 | 14.9 | 9 (4+5) | 5 | + | + | T4 | LOW | ADT | 1,95 | HLA-A\*02 | HLA-A\*25 |
| M28 | 71 | 4.1 | 8 (4+4) | 4 | + | + | T4 | LOW | ADT | 1,36 | HLA-A\*01 | HLA-A\*32 |
| M29 | 68 | 20.0 | 9 (5+4) | 5 | + | + | T4 | LOW | ADT | 4,64 | HLA-A\*02 | HLA-A\*02 |
| M30 | 73 | MISSING | 9 (5+4) | 5 | + | + | T4 | HIGH | ADT | 9,41 | HLA-A\*02 | HLA-A\*23 |
| M31 | 64 | 10.0 | 10 (5+5) | 5 | + | + | T4 | LOW | ADT | 2,22 | HLA-A\*02 | HLA-A\*02 |
| M32 | 68 | 35.0 | 8 (4+4) | 4 | + | + | T4 | HIGH | ADT | 3,99 | HLA-A\*01 | HLA-A\*69 |
| M33 | 80 | 11.0 | 8 (4+4) | 4 | + | + | T4 | HIGH | ADT | 4,67 | HLA-A\*01 | HLA-A\*03 |
| M34 | 67 | 342.0 | MISSING | MISSING | MISSING | + | T4 | HIGH | ADT | 0,80 | HLA-A\*26 | HLA-A\*03 |
| M35 | 66 | 38.3 | 8 (4+4) | 4 | + | + | T4 | LOW | ADT | 5,02 | HLA-A\*02:01 | HLA-A\*26:01 |
| M36 | 61 | 34.8 | 7 (3+4) | 2 | + | + | T4 | LOW | ADT | (9,86) | HLA-A\*33:01 | HLA-A\*24:02 |
| M37 | 69 | 183.0 | 6 (3+3) | 1 | + | + | T4 | HIGH | ADT | (9,90) | HLA-A\*03:01 | HLA-A\*68:01 |
| M38 | 80 | 134.3 | 9 (4+5) | 5 | + | + | T4 | LOW | ADT | 4,67 | HLA-A\*03:01 | HLA-A\*03:01 |
| M39 | 56 | 9.0 | 9 (4+5) | 5 | + | + | T4 | LOW | ADT | 0,46 | HLA-A\*02:01 | HLA-A\*02:01 |
| M40 | 65 | 100.0 | 7 (4+3) | 3 | - | + | T4 | HIGH | ADT | (2,76) | HLA-A\*11:01 | HLA-A\*24:07 |
| M41 | 78 | 388.4 | 9 (5+4) | 5 | + | + | T4 | LOW | ADT | (2,30) | HLA-A\*33:03 | HLA-A\*03:01 |
| M42 | 71 | 126.7 | 8 (4+4) | 4 | + | + | T4 | HIGH | ADT | 3,14 | HLA-A\*24:02 | HLA-A\*24:02 |
| M43 | 49 | 72.7 | 9 (4+5) | 5 | + | + | T4 | HIGH | ADT | 6,48 | HLA-A\*02:02 | HLA-A\*23:01 |
| M44 | 67 | 70.1 | 7 (4+3) | 3 | + | + | T4 | HIGH | ADT | (4,81) | HLA-A\*29:01 | HLA-A\*24:02 |
| M45 | 81 | MISSING | 9 (5+4) | 5 | + | + | T4 | HIGH | ADT | (2,14) | HLA-A\*03:01 | HLA-A\*24:02 |
| M46 | 70 | 841.0 | 9 (4+5) | 5 | + | + | T4 | HIGH | ADT | (2,09) | HLA-A\*03:01 | HLA-A\*24:02 |
| M47 | 88 | 5.5 | 8 (4+4) | 4 | + | + | T4 | LOW | ADT | 4,71 | HLA-A\*26:01 | HLA-A\*24:02 |
| M48 | 84 | 6806.0 | 10 (5+5) | 5 | + | + | T4 | HIGH | ADT | 1,91 | HLA-A\*01:01 | HLA-A\*68:01 |
| M49 | 65 | 2.4 | 9 | 5 | + | + | T4 | HIGH | ADT | 3,75 | HLA-A\*03:01 | HLA-A\*68:01 |
| M50 | 88 | 740.3 | 9 (5+4) | 5 | + | + | T4 | HIGH | ADT | (1,49) | HLA-A\*01:01 | HLA-A\*24:02 |
| M51 | 64 | 183.6 | 9 (5+4) | 5 | + | + | T4 | HIGH | ADT | (1,40) | HLA-A\*02:01 | HLA-A\*01:01 |
| M52 | 67 | 7.0 | 10 (5+5) | 5 | + | + | T4 | HIGH | ADT | (2,74) | HLA-A\*02:01 | HLA-A\*30:01 |
| M54 | 80 | MISSING | 7 (4+3) | 3 | - | + | T4 | HIGH | ADT | 6,49 | HLA-A\*02:01 | HLA-A\*32:01 |
| M55 | 80 | 126.6 | 9 (4+5) | 5 | + | + | T4 | LOW | ADT | (0,97 | HLA-A\*02:01 | HLA-A\*32:01 |
| M56 | 71 | 81.7 | 9 (4+5) | 5 | + | + | T4 | LOW | ADT | (3,78) | HLA-A\*31:01 | HLA-A\*01:01 |
| M57 | 84 | 14.0 | 7 (4+3) | 3 | + | + | T4 | LOW | ADT | (9,74) | HLA-A\*01:01 | HLA-A\*24:02 |
| M58 | 68 | 48.1 | 7 (4+3) | 3 | + | + | T4 | LOW | ADT | (14,01) | HLA-A\*29:01 | HLA-A\*24:02 |
| A17 | 74 | 10.1 | 6 (3+3) | 1 | - | - | T1c | N/A | PRTX | (5,30) | HLA-A\*02:01 | HLA-A\*68:01 |
#
Parentheses indicate censoring of the data at the last time the patient was known not to present the event.
a As reported by Computed Tomography (CT) scan (abdominal) or Whole Body Bone Scan (WBS)
PCa: Prostate cancer, OS: overall survival, ISUP grade group: International Society of Urological Pathology, RP: Radical prostatectomy, PRTX: Primary radiotherapy, ADT: Androgen deprivation therapy, N/A: not applicable.

## Slide 2
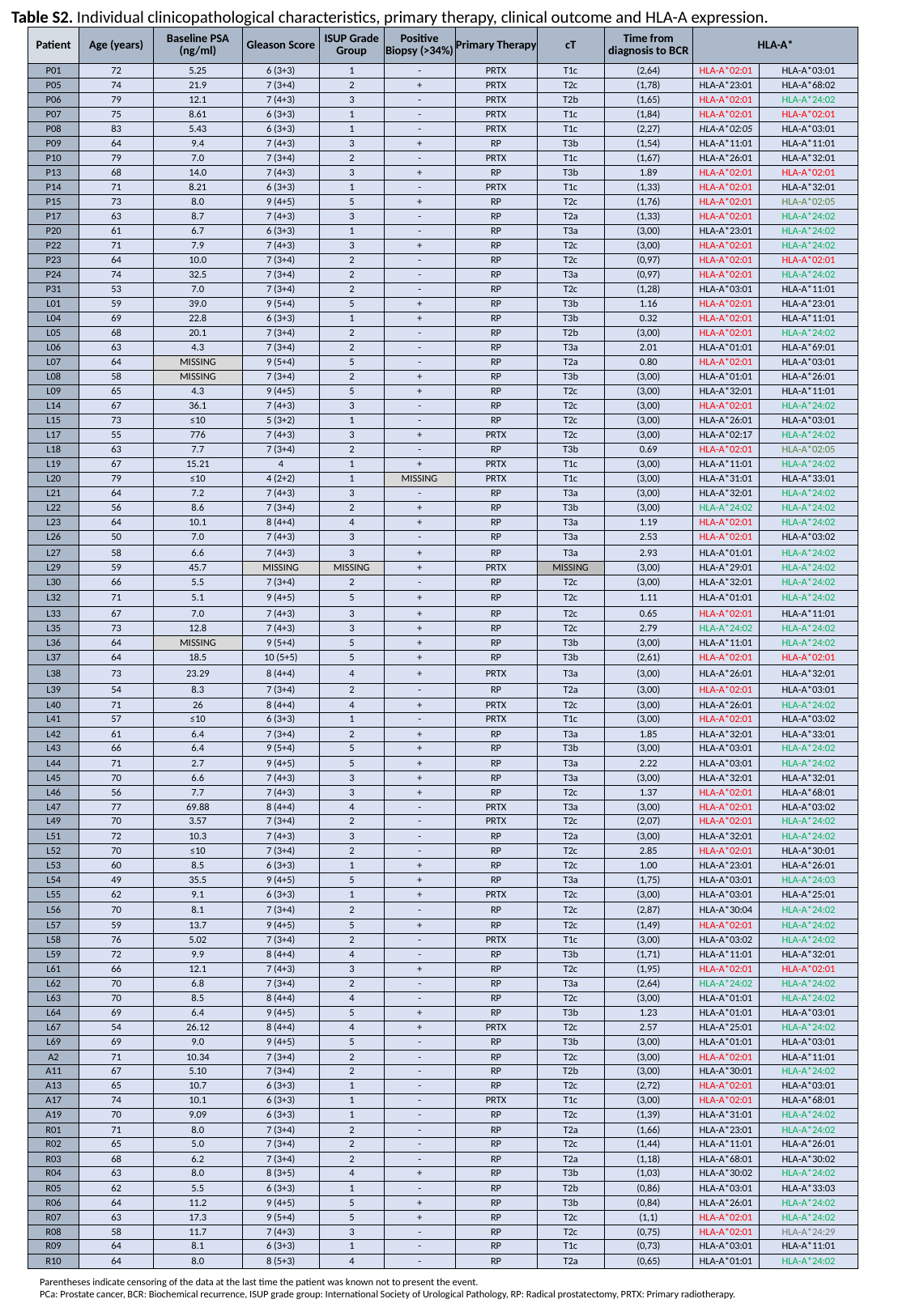

Table S2. Individual clinicopathological characteristics, primary therapy, clinical outcome and HLA-A expression.
| Patient | Age (years) | Baseline PSA (ng/ml) | Gleason Score | ISUP Grade Group | Positive Biopsy (>34%) | Primary Therapy | cT | Time from diagnosis to BCR | HLA-A\* | |
| --- | --- | --- | --- | --- | --- | --- | --- | --- | --- | --- |
| P01 | 72 | 5.25 | 6 (3+3) | 1 | - | PRTX | T1c | (2,64) | HLA-A\*02:01 | HLA-A\*03:01 |
| P05 | 74 | 21.9 | 7 (3+4) | 2 | + | PRTX | T2c | (1,78) | HLA-A\*23:01 | HLA-A\*68:02 |
| P06 | 79 | 12.1 | 7 (4+3) | 3 | - | PRTX | T2b | (1,65) | HLA-A\*02:01 | HLA-A\*24:02 |
| P07 | 75 | 8.61 | 6 (3+3) | 1 | - | PRTX | T1c | (1,84) | HLA-A\*02:01 | HLA-A\*02:01 |
| P08 | 83 | 5.43 | 6 (3+3) | 1 | - | PRTX | T1c | (2,27) | HLA-A\*02:05 | HLA-A\*03:01 |
| P09 | 64 | 9.4 | 7 (4+3) | 3 | + | RP | T3b | (1,54) | HLA-A\*11:01 | HLA-A\*11:01 |
| P10 | 79 | 7.0 | 7 (3+4) | 2 | - | PRTX | T1c | (1,67) | HLA-A\*26:01 | HLA-A\*32:01 |
| P13 | 68 | 14.0 | 7 (4+3) | 3 | + | RP | T3b | 1.89 | HLA-A\*02:01 | HLA-A\*02:01 |
| P14 | 71 | 8.21 | 6 (3+3) | 1 | - | PRTX | T1c | (1,33) | HLA-A\*02:01 | HLA-A\*32:01 |
| P15 | 73 | 8.0 | 9 (4+5) | 5 | + | RP | T2c | (1,76) | HLA-A\*02:01 | HLA-A\*02:05 |
| P17 | 63 | 8.7 | 7 (4+3) | 3 | - | RP | T2a | (1,33) | HLA-A\*02:01 | HLA-A\*24:02 |
| P20 | 61 | 6.7 | 6 (3+3) | 1 | - | RP | T3a | (3,00) | HLA-A\*23:01 | HLA-A\*24:02 |
| P22 | 71 | 7.9 | 7 (4+3) | 3 | + | RP | T2c | (3,00) | HLA-A\*02:01 | HLA-A\*24:02 |
| P23 | 64 | 10.0 | 7 (3+4) | 2 | - | RP | T2c | (0,97) | HLA-A\*02:01 | HLA-A\*02:01 |
| P24 | 74 | 32.5 | 7 (3+4) | 2 | - | RP | T3a | (0,97) | HLA-A\*02:01 | HLA-A\*24:02 |
| P31 | 53 | 7.0 | 7 (3+4) | 2 | - | RP | T2c | (1,28) | HLA-A\*03:01 | HLA-A\*11:01 |
| L01 | 59 | 39.0 | 9 (5+4) | 5 | + | RP | T3b | 1.16 | HLA-A\*02:01 | HLA-A\*23:01 |
| L04 | 69 | 22.8 | 6 (3+3) | 1 | + | RP | T3b | 0.32 | HLA-A\*02:01 | HLA-A\*11:01 |
| L05 | 68 | 20.1 | 7 (3+4) | 2 | - | RP | T2b | (3,00) | HLA-A\*02:01 | HLA-A\*24:02 |
| L06 | 63 | 4.3 | 7 (3+4) | 2 | - | RP | T3a | 2.01 | HLA-A\*01:01 | HLA-A\*69:01 |
| L07 | 64 | MISSING | 9 (5+4) | 5 | - | RP | T2a | 0.80 | HLA-A\*02:01 | HLA-A\*03:01 |
| L08 | 58 | MISSING | 7 (3+4) | 2 | + | RP | T3b | (3,00) | HLA-A\*01:01 | HLA-A\*26:01 |
| L09 | 65 | 4.3 | 9 (4+5) | 5 | + | RP | T2c | (3,00) | HLA-A\*32:01 | HLA-A\*11:01 |
| L14 | 67 | 36.1 | 7 (4+3) | 3 | - | RP | T2c | (3,00) | HLA-A\*02:01 | HLA-A\*24:02 |
| L15 | 73 | ≤10 | 5 (3+2) | 1 | - | RP | T2c | (3,00) | HLA-A\*26:01 | HLA-A\*03:01 |
| L17 | 55 | 776 | 7 (4+3) | 3 | + | PRTX | T2c | (3,00) | HLA-A\*02:17 | HLA-A\*24:02 |
| L18 | 63 | 7.7 | 7 (3+4) | 2 | - | RP | T3b | 0.69 | HLA-A\*02:01 | HLA-A\*02:05 |
| L19 | 67 | 15.21 | 4 | 1 | + | PRTX | T1c | (3,00) | HLA-A\*11:01 | HLA-A\*24:02 |
| L20 | 79 | ≤10 | 4 (2+2) | 1 | MISSING | PRTX | T1c | (3,00) | HLA-A\*31:01 | HLA-A\*33:01 |
| L21 | 64 | 7.2 | 7 (4+3) | 3 | - | RP | T3a | (3,00) | HLA-A\*32:01 | HLA-A\*24:02 |
| L22 | 56 | 8.6 | 7 (3+4) | 2 | + | RP | T3b | (3,00) | HLA-A\*24:02 | HLA-A\*24:02 |
| L23 | 64 | 10.1 | 8 (4+4) | 4 | + | RP | T3a | 1.19 | HLA-A\*02:01 | HLA-A\*24:02 |
| L26 | 50 | 7.0 | 7 (4+3) | 3 | - | RP | T3a | 2.53 | HLA-A\*02:01 | HLA-A\*03:02 |
| L27 | 58 | 6.6 | 7 (4+3) | 3 | + | RP | T3a | 2.93 | HLA-A\*01:01 | HLA-A\*24:02 |
| L29 | 59 | 45.7 | MISSING | MISSING | + | PRTX | MISSING | (3,00) | HLA-A\*29:01 | HLA-A\*24:02 |
| L30 | 66 | 5.5 | 7 (3+4) | 2 | - | RP | T2c | (3,00) | HLA-A\*32:01 | HLA-A\*24:02 |
| L32 | 71 | 5.1 | 9 (4+5) | 5 | + | RP | T2c | 1.11 | HLA-A\*01:01 | HLA-A\*24:02 |
| L33 | 67 | 7.0 | 7 (4+3) | 3 | + | RP | T2c | 0.65 | HLA-A\*02:01 | HLA-A\*11:01 |
| L35 | 73 | 12.8 | 7 (4+3) | 3 | + | RP | T2c | 2.79 | HLA-A\*24:02 | HLA-A\*24:02 |
| L36 | 64 | MISSING | 9 (5+4) | 5 | + | RP | T3b | (3,00) | HLA-A\*11:01 | HLA-A\*24:02 |
| L37 | 64 | 18.5 | 10 (5+5) | 5 | + | RP | T3b | (2,61) | HLA-A\*02:01 | HLA-A\*02:01 |
| L38 | 73 | 23.29 | 8 (4+4) | 4 | + | PRTX | T3a | (3,00) | HLA-A\*26:01 | HLA-A\*32:01 |
| L39 | 54 | 8.3 | 7 (3+4) | 2 | - | RP | T2a | (3,00) | HLA-A\*02:01 | HLA-A\*03:01 |
| L40 | 71 | 26 | 8 (4+4) | 4 | + | PRTX | T2c | (3,00) | HLA-A\*26:01 | HLA-A\*24:02 |
| L41 | 57 | ≤10 | 6 (3+3) | 1 | - | PRTX | T1c | (3,00) | HLA-A\*02:01 | HLA-A\*03:02 |
| L42 | 61 | 6.4 | 7 (3+4) | 2 | + | RP | T3a | 1.85 | HLA-A\*32:01 | HLA-A\*33:01 |
| L43 | 66 | 6.4 | 9 (5+4) | 5 | + | RP | T3b | (3,00) | HLA-A\*03:01 | HLA-A\*24:02 |
| L44 | 71 | 2.7 | 9 (4+5) | 5 | + | RP | T3a | 2.22 | HLA-A\*03:01 | HLA-A\*24:02 |
| L45 | 70 | 6.6 | 7 (4+3) | 3 | + | RP | T3a | (3,00) | HLA-A\*32:01 | HLA-A\*32:01 |
| L46 | 56 | 7.7 | 7 (4+3) | 3 | + | RP | T2c | 1.37 | HLA-A\*02:01 | HLA-A\*68:01 |
| L47 | 77 | 69.88 | 8 (4+4) | 4 | - | PRTX | T3a | (3,00) | HLA-A\*02:01 | HLA-A\*03:02 |
| L49 | 70 | 3.57 | 7 (3+4) | 2 | - | PRTX | T2c | (2,07) | HLA-A\*02:01 | HLA-A\*24:02 |
| L51 | 72 | 10.3 | 7 (4+3) | 3 | - | RP | T2a | (3,00) | HLA-A\*32:01 | HLA-A\*24:02 |
| L52 | 70 | ≤10 | 7 (3+4) | 2 | - | RP | T2c | 2.85 | HLA-A\*02:01 | HLA-A\*30:01 |
| L53 | 60 | 8.5 | 6 (3+3) | 1 | + | RP | T2c | 1.00 | HLA-A\*23:01 | HLA-A\*26:01 |
| L54 | 49 | 35.5 | 9 (4+5) | 5 | + | RP | T3a | (1,75) | HLA-A\*03:01 | HLA-A\*24:03 |
| L55 | 62 | 9.1 | 6 (3+3) | 1 | + | PRTX | T2c | (3,00) | HLA-A\*03:01 | HLA-A\*25:01 |
| L56 | 70 | 8.1 | 7 (3+4) | 2 | - | RP | T2c | (2,87) | HLA-A\*30:04 | HLA-A\*24:02 |
| L57 | 59 | 13.7 | 9 (4+5) | 5 | + | RP | T2c | (1,49) | HLA-A\*02:01 | HLA-A\*24:02 |
| L58 | 76 | 5.02 | 7 (3+4) | 2 | - | PRTX | T1c | (3,00) | HLA-A\*03:02 | HLA-A\*24:02 |
| L59 | 72 | 9.9 | 8 (4+4) | 4 | - | RP | T3b | (1,71) | HLA-A\*11:01 | HLA-A\*32:01 |
| L61 | 66 | 12.1 | 7 (4+3) | 3 | + | RP | T2c | (1,95) | HLA-A\*02:01 | HLA-A\*02:01 |
| L62 | 70 | 6.8 | 7 (3+4) | 2 | - | RP | T3a | (2,64) | HLA-A\*24:02 | HLA-A\*24:02 |
| L63 | 70 | 8.5 | 8 (4+4) | 4 | - | RP | T2c | (3,00) | HLA-A\*01:01 | HLA-A\*24:02 |
| L64 | 69 | 6.4 | 9 (4+5) | 5 | + | RP | T3b | 1.23 | HLA-A\*01:01 | HLA-A\*03:01 |
| L67 | 54 | 26.12 | 8 (4+4) | 4 | + | PRTX | T2c | 2.57 | HLA-A\*25:01 | HLA-A\*24:02 |
| L69 | 69 | 9.0 | 9 (4+5) | 5 | - | RP | T3b | (3,00) | HLA-A\*01:01 | HLA-A\*03:01 |
| A2 | 71 | 10.34 | 7 (3+4) | 2 | - | RP | T2c | (3,00) | HLA-A\*02:01 | HLA-A\*11:01 |
| A11 | 67 | 5.10 | 7 (3+4) | 2 | - | RP | T2b | (3,00) | HLA-A\*30:01 | HLA-A\*24:02 |
| A13 | 65 | 10.7 | 6 (3+3) | 1 | - | RP | T2c | (2,72) | HLA-A\*02:01 | HLA-A\*03:01 |
| A17 | 74 | 10.1 | 6 (3+3) | 1 | - | PRTX | T1c | (3,00) | HLA-A\*02:01 | HLA-A\*68:01 |
| A19 | 70 | 9.09 | 6 (3+3) | 1 | - | RP | T2c | (1,39) | HLA-A\*31:01 | HLA-A\*24:02 |
| R01 | 71 | 8.0 | 7 (3+4) | 2 | - | RP | T2a | (1,66) | HLA-A\*23:01 | HLA-A\*24:02 |
| R02 | 65 | 5.0 | 7 (3+4) | 2 | - | RP | T2c | (1,44) | HLA-A\*11:01 | HLA-A\*26:01 |
| R03 | 68 | 6.2 | 7 (3+4) | 2 | - | RP | T2a | (1,18) | HLA-A\*68:01 | HLA-A\*30:02 |
| R04 | 63 | 8.0 | 8 (3+5) | 4 | + | RP | T3b | (1,03) | HLA-A\*30:02 | HLA-A\*24:02 |
| R05 | 62 | 5.5 | 6 (3+3) | 1 | - | RP | T2b | (0,86) | HLA-A\*03:01 | HLA-A\*33:03 |
| R06 | 64 | 11.2 | 9 (4+5) | 5 | + | RP | T3b | (0,84) | HLA-A\*26:01 | HLA-A\*24:02 |
| R07 | 63 | 17.3 | 9 (5+4) | 5 | + | RP | T2c | (1,1) | HLA-A\*02:01 | HLA-A\*24:02 |
| R08 | 58 | 11.7 | 7 (4+3) | 3 | - | RP | T2c | (0,75) | HLA-A\*02:01 | HLA-A\*24:29 |
| R09 | 64 | 8.1 | 6 (3+3) | 1 | - | RP | T1c | (0,73) | HLA-A\*03:01 | HLA-A\*11:01 |
| R10 | 64 | 8.0 | 8 (5+3) | 4 | - | RP | T2a | (0,65) | HLA-A\*01:01 | HLA-A\*24:02 |
Parentheses indicate censoring of the data at the last time the patient was known not to present the event.
PCa: Prostate cancer, BCR: Biochemical recurrence, ISUP grade group: International Society of Urological Pathology, RP: Radical prostatectomy, PRTX: Primary radiotherapy.

## Slide 3
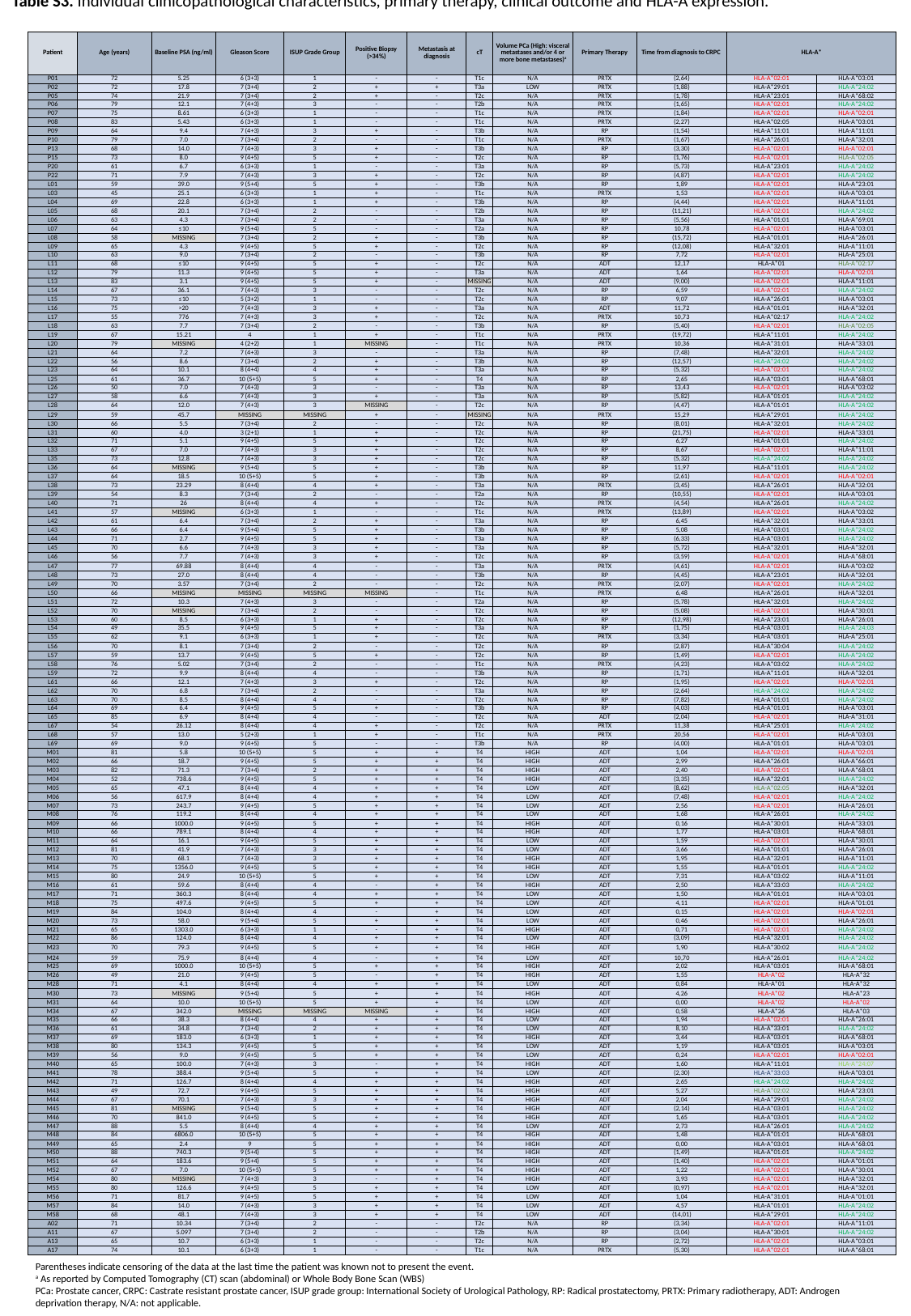

Table S3. Individual clinicopathological characteristics, primary therapy, clinical outcome and HLA-A expression.
| Patient | Age (years) | Baseline PSA (ng/ml) | Gleason Score | ISUP Grade Group | Positive Biopsy (>34%) | Metastasis at diagnosis | cT | Volume PCa (High: visceral metastases and/or 4 or more bone metastases)a | Primary Therapy | Time from diagnosis to CRPC | HLA-A\* | |
| --- | --- | --- | --- | --- | --- | --- | --- | --- | --- | --- | --- | --- |
| P01 | 72 | 5.25 | 6 (3+3) | 1 | - | - | T1c | N/A | PRTX | (2,64) | HLA-A\*02:01 | HLA-A\*03:01 |
| P02 | 72 | 17.8 | 7 (3+4) | 2 | + | + | T3a | LOW | PRTX | (1,88) | HLA-A\*29:01 | HLA-A\*24:02 |
| P05 | 74 | 21.9 | 7 (3+4) | 2 | + | - | T2c | N/A | PRTX | (1,78) | HLA-A\*23:01 | HLA-A\*68:02 |
| P06 | 79 | 12.1 | 7 (4+3) | 3 | - | - | T2b | N/A | PRTX | (1,65) | HLA-A\*02:01 | HLA-A\*24:02 |
| P07 | 75 | 8.61 | 6 (3+3) | 1 | - | - | T1c | N/A | PRTX | (1,84) | HLA-A\*02:01 | HLA-A\*02:01 |
| P08 | 83 | 5.43 | 6 (3+3) | 1 | - | - | T1c | N/A | PRTX | (2,27) | HLA-A\*02:05 | HLA-A\*03:01 |
| P09 | 64 | 9.4 | 7 (4+3) | 3 | + | - | T3b | N/A | RP | (1,54) | HLA-A\*11:01 | HLA-A\*11:01 |
| P10 | 79 | 7.0 | 7 (3+4) | 2 | - | - | T1c | N/A | PRTX | (1,67) | HLA-A\*26:01 | HLA-A\*32:01 |
| P13 | 68 | 14.0 | 7 (4+3) | 3 | + | - | T3b | N/A | RP | (3,30) | HLA-A\*02:01 | HLA-A\*02:01 |
| P15 | 73 | 8.0 | 9 (4+5) | 5 | + | - | T2c | N/A | RP | (1,76) | HLA-A\*02:01 | HLA-A\*02:05 |
| P20 | 61 | 6.7 | 6 (3+3) | 1 | - | - | T3a | N/A | RP | (5,73) | HLA-A\*23:01 | HLA-A\*24:02 |
| P22 | 71 | 7.9 | 7 (4+3) | 3 | + | - | T2c | N/A | RP | (4,87) | HLA-A\*02:01 | HLA-A\*24:02 |
| L01 | 59 | 39.0 | 9 (5+4) | 5 | + | - | T3b | N/A | RP | 1,89 | HLA-A\*02:01 | HLA-A\*23:01 |
| L03 | 45 | 25.1 | 6 (3+3) | 1 | + | - | T1c | N/A | PRTX | 1,53 | HLA-A\*02:01 | HLA-A\*03:01 |
| L04 | 69 | 22.8 | 6 (3+3) | 1 | + | - | T3b | N/A | RP | (4,44) | HLA-A\*02:01 | HLA-A\*11:01 |
| L05 | 68 | 20.1 | 7 (3+4) | 2 | - | - | T2b | N/A | RP | (11,21) | HLA-A\*02:01 | HLA-A\*24:02 |
| L06 | 63 | 4.3 | 7 (3+4) | 2 | - | - | T3a | N/A | RP | (5,56) | HLA-A\*01:01 | HLA-A\*69:01 |
| L07 | 64 | ≤10 | 9 (5+4) | 5 | - | - | T2a | N/A | RP | 10,78 | HLA-A\*02:01 | HLA-A\*03:01 |
| L08 | 58 | MISSING | 7 (3+4) | 2 | + | - | T3b | N/A | RP | (15,72) | HLA-A\*01:01 | HLA-A\*26:01 |
| L09 | 65 | 4.3 | 9 (4+5) | 5 | + | - | T2c | N/A | RP | (12,08) | HLA-A\*32:01 | HLA-A\*11:01 |
| L10 | 63 | 9.0 | 7 (3+4) | 2 | - | - | T3b | N/A | RP | 7,72 | HLA-A\*02:01 | HLA-A\*25:01 |
| L11 | 68 | ≤10 | 9 (4+5) | 5 | + | - | T2c | N/A | ADT | 12,17 | HLA-A\*01 | HLA-A\*02:17 |
| L12 | 79 | 11.3 | 9 (4+5) | 5 | + | - | T3a | N/A | ADT | 1,64 | HLA-A\*02:01 | HLA-A\*02:01 |
| L13 | 83 | 3.1 | 9 (4+5) | 5 | + | - | MISSING | N/A | ADT | (9,00) | HLA-A\*02:01 | HLA-A\*11:01 |
| L14 | 67 | 36.1 | 7 (4+3) | 3 | - | - | T2c | N/A | RP | 6,59 | HLA-A\*02:01 | HLA-A\*24:02 |
| L15 | 73 | ≤10 | 5 (3+2) | 1 | - | - | T2c | N/A | RP | 9,07 | HLA-A\*26:01 | HLA-A\*03:01 |
| L16 | 75 | >20 | 7 (4+3) | 3 | + | - | T3a | N/A | ADT | 11,72 | HLA-A\*01:01 | HLA-A\*32:01 |
| L17 | 55 | 776 | 7 (4+3) | 3 | + | - | T2c | N/A | PRTX | 10,73 | HLA-A\*02:17 | HLA-A\*24:02 |
| L18 | 63 | 7.7 | 7 (3+4) | 2 | - | - | T3b | N/A | RP | (5,40) | HLA-A\*02:01 | HLA-A\*02:05 |
| L19 | 67 | 15.21 | 4 | 1 | + | - | T1c | N/A | PRTX | (19,72) | HLA-A\*11:01 | HLA-A\*24:02 |
| L20 | 79 | MISSING | 4 (2+2) | 1 | MISSING | - | T1c | N/A | PRTX | 10,36 | HLA-A\*31:01 | HLA-A\*33:01 |
| L21 | 64 | 7.2 | 7 (4+3) | 3 | - | - | T3a | N/A | RP | (7,48) | HLA-A\*32:01 | HLA-A\*24:02 |
| L22 | 56 | 8.6 | 7 (3+4) | 2 | + | - | T3b | N/A | RP | (12,57) | HLA-A\*24:02 | HLA-A\*24:02 |
| L23 | 64 | 10.1 | 8 (4+4) | 4 | + | - | T3a | N/A | RP | (5,32) | HLA-A\*02:01 | HLA-A\*24:02 |
| L25 | 61 | 36.7 | 10 (5+5) | 5 | + | - | T4 | N/A | RP | 2,65 | HLA-A\*03:01 | HLA-A\*68:01 |
| L26 | 50 | 7.0 | 7 (4+3) | 3 | - | - | T3a | N/A | RP | 13,43 | HLA-A\*02:01 | HLA-A\*03:02 |
| L27 | 58 | 6.6 | 7 (4+3) | 3 | + | - | T3a | N/A | RP | (5,82) | HLA-A\*01:01 | HLA-A\*24:02 |
| L28 | 64 | 12.0 | 7 (4+3) | 3 | MISSING | - | T2c | N/A | RP | (4,47) | HLA-A\*01:01 | HLA-A\*24:02 |
| L29 | 59 | 45.7 | MISSING | MISSING | + | - | MISSING | N/A | PRTX | 15,29 | HLA-A\*29:01 | HLA-A\*24:02 |
| L30 | 66 | 5.5 | 7 (3+4) | 2 | - | - | T2c | N/A | RP | (8,01) | HLA-A\*32:01 | HLA-A\*24:02 |
| L31 | 60 | 4.0 | 3 (2+1) | 1 | + | - | T2c | N/A | RP | (21,75) | HLA-A\*02:01 | HLA-A\*33:01 |
| L32 | 71 | 5.1 | 9 (4+5) | 5 | + | - | T2c | N/A | RP | 6,27 | HLA-A\*01:01 | HLA-A\*24:02 |
| L33 | 67 | 7.0 | 7 (4+3) | 3 | + | - | T2c | N/A | RP | 8,67 | HLA-A\*02:01 | HLA-A\*11:01 |
| L35 | 73 | 12.8 | 7 (4+3) | 3 | + | - | T2c | N/A | RP | (5,32) | HLA-A\*24:02 | HLA-A\*24:02 |
| L36 | 64 | MISSING | 9 (5+4) | 5 | + | - | T3b | N/A | RP | 11,97 | HLA-A\*11:01 | HLA-A\*24:02 |
| L37 | 64 | 18.5 | 10 (5+5) | 5 | + | - | T3b | N/A | RP | (2,61) | HLA-A\*02:01 | HLA-A\*02:01 |
| L38 | 73 | 23.29 | 8 (4+4) | 4 | + | - | T3a | N/A | PRTX | (3,45) | HLA-A\*26:01 | HLA-A\*32:01 |
| L39 | 54 | 8.3 | 7 (3+4) | 2 | - | - | T2a | N/A | RP | (10,55) | HLA-A\*02:01 | HLA-A\*03:01 |
| L40 | 71 | 26 | 8 (4+4) | 4 | + | - | T2c | N/A | PRTX | (4,54) | HLA-A\*26:01 | HLA-A\*24:02 |
| L41 | 57 | MISSING | 6 (3+3) | 1 | - | - | T1c | N/A | PRTX | (13,89) | HLA-A\*02:01 | HLA-A\*03:02 |
| L42 | 61 | 6.4 | 7 (3+4) | 2 | + | - | T3a | N/A | RP | 6,45 | HLA-A\*32:01 | HLA-A\*33:01 |
| L43 | 66 | 6.4 | 9 (5+4) | 5 | + | - | T3b | N/A | RP | 5,08 | HLA-A\*03:01 | HLA-A\*24:02 |
| L44 | 71 | 2.7 | 9 (4+5) | 5 | + | - | T3a | N/A | RP | (6,33) | HLA-A\*03:01 | HLA-A\*24:02 |
| L45 | 70 | 6.6 | 7 (4+3) | 3 | + | - | T3a | N/A | RP | (5,72) | HLA-A\*32:01 | HLA-A\*32:01 |
| L46 | 56 | 7.7 | 7 (4+3) | 3 | + | - | T2c | N/A | RP | (3,59) | HLA-A\*02:01 | HLA-A\*68:01 |
| L47 | 77 | 69.88 | 8 (4+4) | 4 | - | - | T3a | N/A | PRTX | (4,61) | HLA-A\*02:01 | HLA-A\*03:02 |
| L48 | 73 | 27.0 | 8 (4+4) | 4 | - | - | T3b | N/A | RP | (4,45) | HLA-A\*23:01 | HLA-A\*32:01 |
| L49 | 70 | 3.57 | 7 (3+4) | 2 | - | - | T2c | N/A | PRTX | (2,07) | HLA-A\*02:01 | HLA-A\*24:02 |
| L50 | 66 | MISSING | MISSING | MISSING | MISSING | - | T1c | N/A | PRTX | 6,48 | HLA-A\*26:01 | HLA-A\*32:01 |
| L51 | 72 | 10.3 | 7 (4+3) | 3 | - | - | T2a | N/A | RP | (5,78) | HLA-A\*32:01 | HLA-A\*24:02 |
| L52 | 70 | MISSING | 7 (3+4) | 2 | - | - | T2c | N/A | RP | (5,08) | HLA-A\*02:01 | HLA-A\*30:01 |
| L53 | 60 | 8.5 | 6 (3+3) | 1 | + | - | T2c | N/A | RP | (12,98) | HLA-A\*23:01 | HLA-A\*26:01 |
| L54 | 49 | 35.5 | 9 (4+5) | 5 | + | - | T3a | N/A | RP | (1,75) | HLA-A\*03:01 | HLA-A\*24:03 |
| L55 | 62 | 9.1 | 6 (3+3) | 1 | + | - | T2c | N/A | PRTX | (3,34) | HLA-A\*03:01 | HLA-A\*25:01 |
| L56 | 70 | 8.1 | 7 (3+4) | 2 | - | - | T2c | N/A | RP | (2,87) | HLA-A\*30:04 | HLA-A\*24:02 |
| L57 | 59 | 13.7 | 9 (4+5) | 5 | + | - | T2c | N/A | RP | (1,49) | HLA-A\*02:01 | HLA-A\*24:02 |
| L58 | 76 | 5.02 | 7 (3+4) | 2 | - | - | T1c | N/A | PRTX | (4,23) | HLA-A\*03:02 | HLA-A\*24:02 |
| L59 | 72 | 9.9 | 8 (4+4) | 4 | - | - | T3b | N/A | RP | (1,71) | HLA-A\*11:01 | HLA-A\*32:01 |
| L61 | 66 | 12.1 | 7 (4+3) | 3 | + | - | T2c | N/A | RP | (1,95) | HLA-A\*02:01 | HLA-A\*02:01 |
| L62 | 70 | 6.8 | 7 (3+4) | 2 | - | - | T3a | N/A | RP | (2,64) | HLA-A\*24:02 | HLA-A\*24:02 |
| L63 | 70 | 8.5 | 8 (4+4) | 4 | - | - | T2c | N/A | RP | (7,82) | HLA-A\*01:01 | HLA-A\*24:02 |
| L64 | 69 | 6.4 | 9 (4+5) | 5 | + | - | T3b | N/A | RP | (4,03) | HLA-A\*01:01 | HLA-A\*03:01 |
| L65 | 85 | 6.9 | 8 (4+4) | 4 | - | - | T2c | N/A | ADT | (2,04) | HLA-A\*02:01 | HLA-A\*31:01 |
| L67 | 54 | 26.12 | 8 (4+4) | 4 | + | - | T2c | N/A | PRTX | 11,38 | HLA-A\*25:01 | HLA-A\*24:02 |
| L68 | 57 | 13.0 | 5 (2+3) | 1 | + | - | T1c | N/A | PRTX | 20,56 | HLA-A\*02:01 | HLA-A\*03:01 |
| L69 | 69 | 9.0 | 9 (4+5) | 5 | - | - | T3b | N/A | RP | (4,00) | HLA-A\*01:01 | HLA-A\*03:01 |
| M01 | 81 | 5.8 | 10 (5+5) | 5 | + | + | T4 | HIGH | ADT | 1,04 | HLA-A\*02:01 | HLA-A\*02:01 |
| M02 | 66 | 18.7 | 9 (4+5) | 5 | + | + | T4 | HIGH | ADT | 2,99 | HLA-A\*26:01 | HLA-A\*66:01 |
| M03 | 82 | 71.3 | 7 (3+4) | 2 | + | + | T4 | HIGH | ADT | 2,40 | HLA-A\*02:01 | HLA-A\*68:01 |
| M04 | 52 | 738.6 | 9 (4+5) | 5 | + | + | T4 | HIGH | ADT | (3,35) | HLA-A\*32:01 | HLA-A\*24:02 |
| M05 | 65 | 47.1 | 8 (4+4) | 4 | + | + | T4 | LOW | ADT | (8,62) | HLA-A\*02:05 | HLA-A\*32:01 |
| M06 | 56 | 617.9 | 8 (4+4) | 4 | + | + | T4 | LOW | ADT | (7,48) | HLA-A\*02:01 | HLA-A\*24:02 |
| M07 | 73 | 243.7 | 9 (4+5) | 5 | + | + | T4 | LOW | ADT | 2,56 | HLA-A\*02:01 | HLA-A\*26:01 |
| M08 | 76 | 119.2 | 8 (4+4) | 4 | + | + | T4 | LOW | ADT | 1,68 | HLA-A\*26:01 | HLA-A\*24:02 |
| M09 | 66 | 1000.0 | 9 (4+5) | 5 | + | + | T4 | HIGH | ADT | 0,16 | HLA-A\*30:01 | HLA-A\*33:01 |
| M10 | 66 | 789.1 | 8 (4+4) | 4 | + | + | T4 | HIGH | ADT | 1,77 | HLA-A\*03:01 | HLA-A\*68:01 |
| M11 | 64 | 16.1 | 9 (4+5) | 5 | + | + | T4 | LOW | ADT | 1,59 | HLA-A\*02:01 | HLA-A\*30:01 |
| M12 | 81 | 41.9 | 7 (4+3) | 3 | + | + | T4 | LOW | ADT | 3,66 | HLA-A\*01:01 | HLA-A\*26:01 |
| M13 | 70 | 68.1 | 7 (4+3) | 3 | + | + | T4 | HIGH | ADT | 1,95 | HLA-A\*32:01 | HLA-A\*11:01 |
| M14 | 75 | 1356.0 | 9 (4+5) | 5 | + | + | T4 | HIGH | ADT | 1,55 | HLA-A\*01:01 | HLA-A\*24:02 |
| M15 | 80 | 24.9 | 10 (5+5) | 5 | + | + | T4 | LOW | ADT | 7,31 | HLA-A\*03:02 | HLA-A\*11:01 |
| M16 | 61 | 59.6 | 8 (4+4) | 4 | - | + | T4 | HIGH | ADT | 2,50 | HLA-A\*33:03 | HLA-A\*24:02 |
| M17 | 71 | 360.3 | 8 (4+4) | 4 | + | + | T4 | LOW | ADT | 1,50 | HLA-A\*01:01 | HLA-A\*03:01 |
| M18 | 75 | 497.6 | 9 (4+5) | 5 | + | + | T4 | LOW | ADT | 4,11 | HLA-A\*02:01 | HLA-A\*01:01 |
| M19 | 84 | 104.0 | 8 (4+4) | 4 | - | + | T4 | LOW | ADT | 0,15 | HLA-A\*02:01 | HLA-A\*02:01 |
| M20 | 73 | 58.0 | 9 (5+4) | 5 | + | + | T4 | LOW | ADT | 0,46 | HLA-A\*02:01 | HLA-A\*26:01 |
| M21 | 65 | 1303.0 | 6 (3+3) | 1 | - | + | T4 | HIGH | ADT | 0,71 | HLA-A\*02:01 | HLA-A\*24:02 |
| M22 | 86 | 124.0 | 8 (4+4) | 4 | + | + | T4 | LOW | ADT | (3,09) | HLA-A\*32:01 | HLA-A\*24:02 |
| M23 | 70 | 79.3 | 9 (4+5) | 5 | + | + | T4 | HIGH | ADT | 1,90 | HLA-A\*30:02 | HLA-A\*24:02 |
| M24 | 59 | 75.9 | 8 (4+4) | 4 | - | + | T4 | LOW | ADT | 10,70 | HLA-A\*26:01 | HLA-A\*24:02 |
| M25 | 69 | 1000.0 | 10 (5+5) | 5 | + | + | T4 | HIGH | ADT | 2,02 | HLA-A\*03:01 | HLA-A\*68:01 |
| M26 | 49 | 21.0 | 9 (4+5) | 5 | - | + | T4 | HIGH | ADT | 1,55 | HLA-A\*02 | HLA-A\*32 |
| M28 | 71 | 4.1 | 8 (4+4) | 4 | + | + | T4 | LOW | ADT | 0,84 | HLA-A\*01 | HLA-A\*32 |
| M30 | 73 | MISSING | 9 (5+4) | 5 | + | + | T4 | HIGH | ADT | 4,26 | HLA-A\*02 | HLA-A\*23 |
| M31 | 64 | 10.0 | 10 (5+5) | 5 | + | + | T4 | LOW | ADT | 0,00 | HLA-A\*02 | HLA-A\*02 |
| M34 | 67 | 342.0 | MISSING | MISSING | MISSING | + | T4 | HIGH | ADT | 0,58 | HLA-A\*26 | HLA-A\*03 |
| M35 | 66 | 38.3 | 8 (4+4) | 4 | + | + | T4 | LOW | ADT | 1,94 | HLA-A\*02:01 | HLA-A\*26:01 |
| M36 | 61 | 34.8 | 7 (3+4) | 2 | + | + | T4 | LOW | ADT | 8,10 | HLA-A\*33:01 | HLA-A\*24:02 |
| M37 | 69 | 183.0 | 6 (3+3) | 1 | + | + | T4 | HIGH | ADT | 3,44 | HLA-A\*03:01 | HLA-A\*68:01 |
| M38 | 80 | 134.3 | 9 (4+5) | 5 | + | + | T4 | LOW | ADT | 1,19 | HLA-A\*03:01 | HLA-A\*03:01 |
| M39 | 56 | 9.0 | 9 (4+5) | 5 | + | + | T4 | LOW | ADT | 0,24 | HLA-A\*02:01 | HLA-A\*02:01 |
| M40 | 65 | 100.0 | 7 (4+3) | 3 | - | + | T4 | HIGH | ADT | 1,60 | HLA-A\*11:01 | HLA-A\*24:07 |
| M41 | 78 | 388.4 | 9 (5+4) | 5 | + | + | T4 | LOW | ADT | (2,30) | HLA-A\*33:03 | HLA-A\*03:01 |
| M42 | 71 | 126.7 | 8 (4+4) | 4 | + | + | T4 | HIGH | ADT | 2,65 | HLA-A\*24:02 | HLA-A\*24:02 |
| M43 | 49 | 72.7 | 9 (4+5) | 5 | + | + | T4 | HIGH | ADT | 5,27 | HLA-A\*02:02 | HLA-A\*23:01 |
| M44 | 67 | 70.1 | 7 (4+3) | 3 | + | + | T4 | HIGH | ADT | 2,04 | HLA-A\*29:01 | HLA-A\*24:02 |
| M45 | 81 | MISSING | 9 (5+4) | 5 | + | + | T4 | HIGH | ADT | (2,14) | HLA-A\*03:01 | HLA-A\*24:02 |
| M46 | 70 | 841.0 | 9 (4+5) | 5 | + | + | T4 | HIGH | ADT | 1,65 | HLA-A\*03:01 | HLA-A\*24:02 |
| M47 | 88 | 5.5 | 8 (4+4) | 4 | + | + | T4 | LOW | ADT | 2,73 | HLA-A\*26:01 | HLA-A\*24:02 |
| M48 | 84 | 6806.0 | 10 (5+5) | 5 | + | + | T4 | HIGH | ADT | 1,48 | HLA-A\*01:01 | HLA-A\*68:01 |
| M49 | 65 | 2.4 | 9 | 5 | + | + | T4 | HIGH | ADT | 0,00 | HLA-A\*03:01 | HLA-A\*68:01 |
| M50 | 88 | 740.3 | 9 (5+4) | 5 | + | + | T4 | HIGH | ADT | (1,49) | HLA-A\*01:01 | HLA-A\*24:02 |
| M51 | 64 | 183.6 | 9 (5+4) | 5 | + | + | T4 | HIGH | ADT | (1,40) | HLA-A\*02:01 | HLA-A\*01:01 |
| M52 | 67 | 7.0 | 10 (5+5) | 5 | + | + | T4 | HIGH | ADT | 1,22 | HLA-A\*02:01 | HLA-A\*30:01 |
| M54 | 80 | MISSING | 7 (4+3) | 3 | - | + | T4 | HIGH | ADT | 3,93 | HLA-A\*02:01 | HLA-A\*32:01 |
| M55 | 80 | 126.6 | 9 (4+5) | 5 | + | + | T4 | LOW | ADT | (0,97) | HLA-A\*02:01 | HLA-A\*32:01 |
| M56 | 71 | 81.7 | 9 (4+5) | 5 | + | + | T4 | LOW | ADT | 1,04 | HLA-A\*31:01 | HLA-A\*01:01 |
| M57 | 84 | 14.0 | 7 (4+3) | 3 | + | + | T4 | LOW | ADT | 4,57 | HLA-A\*01:01 | HLA-A\*24:02 |
| M58 | 68 | 48.1 | 7 (4+3) | 3 | + | + | T4 | LOW | ADT | (14,01) | HLA-A\*29:01 | HLA-A\*24:02 |
| A02 | 71 | 10.34 | 7 (3+4) | 2 | - | - | T2c | N/A | RP | (3,34) | HLA-A\*02:01 | HLA-A\*11:01 |
| A11 | 67 | 5.097 | 7 (3+4) | 2 | - | - | T2b | N/A | RP | (3,04) | HLA-A\*30:01 | HLA-A\*24:02 |
| A13 | 65 | 10.7 | 6 (3+3) | 1 | - | - | T2c | N/A | RP | (2,72) | HLA-A\*02:01 | HLA-A\*03:01 |
| A17 | 74 | 10.1 | 6 (3+3) | 1 | - | - | T1c | N/A | PRTX | (5,30) | HLA-A\*02:01 | HLA-A\*68:01 |
Parentheses indicate censoring of the data at the last time the patient was known not to present the event.
a As reported by Computed Tomography (CT) scan (abdominal) or Whole Body Bone Scan (WBS)
PCa: Prostate cancer, CRPC: Castrate resistant prostate cancer, ISUP grade group: International Society of Urological Pathology, RP: Radical prostatectomy, PRTX: Primary radiotherapy, ADT: Androgen deprivation therapy, N/A: not applicable.

## Slide 4
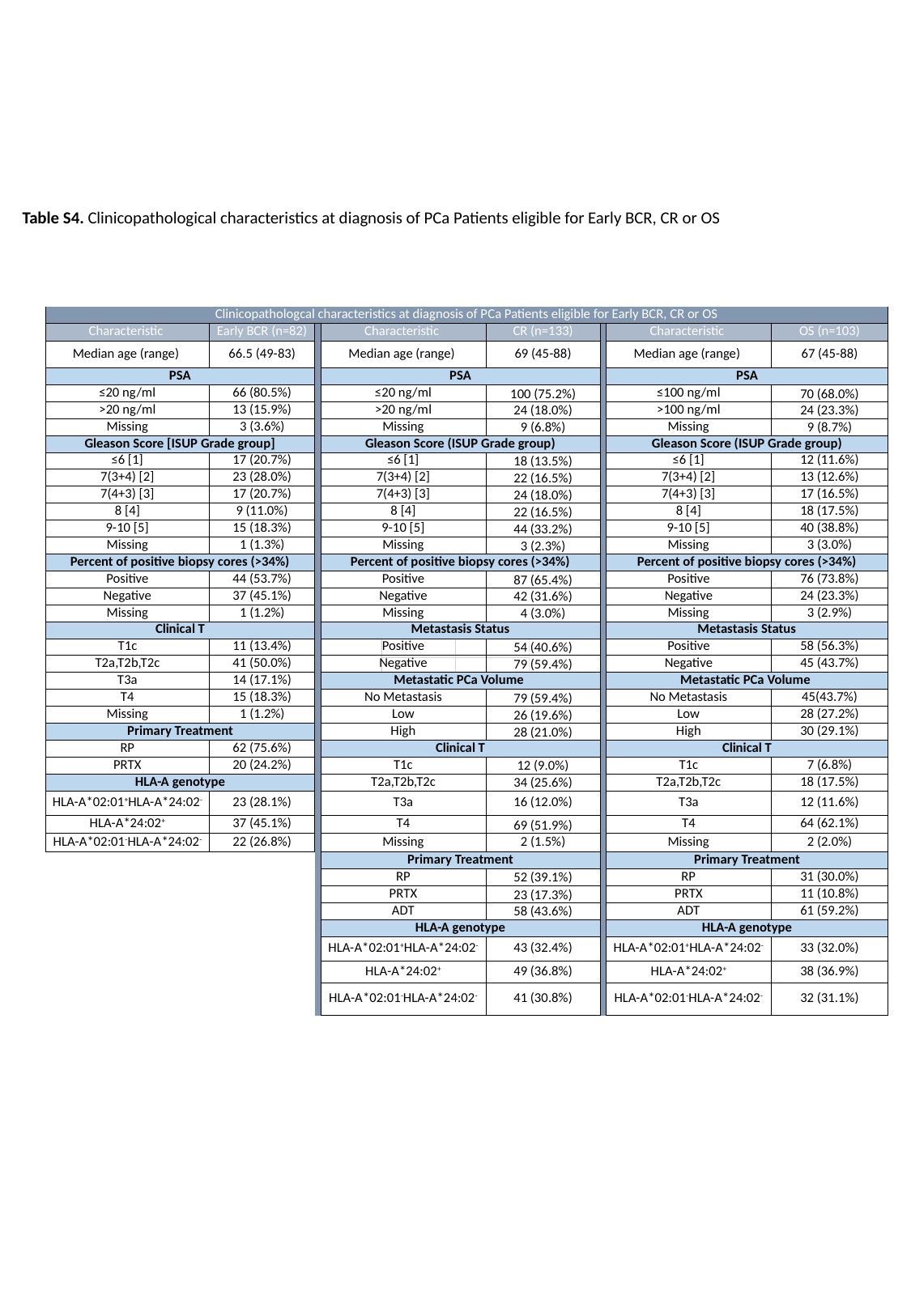

Table S4. Clinicopathological characteristics at diagnosis of PCa Patients eligible for Early BCR, CR or OS
| Clinicopathologcal characteristics at diagnosis of PCa Patients eligible for Early BCR, CR or OS | | | | | | | |
| --- | --- | --- | --- | --- | --- | --- | --- |
| Characteristic | Early BCR (n=82) | | Characteristic | CR (n=133) | | Characteristic | OS (n=103) |
| Median age (range) | 66.5 (49-83) | | Median age (range) | 69 (45-88) | | Median age (range) | 67 (45-88) |
| PSA | | | PSA | | | PSA | |
| ≤20 ng/ml | 66 (80.5%) | | ≤20 ng/ml | 100 (75.2%) | | ≤100 ng/ml | 70 (68.0%) |
| >20 ng/ml | 13 (15.9%) | | >20 ng/ml | 24 (18.0%) | | >100 ng/ml | 24 (23.3%) |
| Missing | 3 (3.6%) | | Missing | 9 (6.8%) | | Missing | 9 (8.7%) |
| Gleason Score [ISUP Grade group] | | | Gleason Score (ISUP Grade group) | | | Gleason Score (ISUP Grade group) | |
| ≤6 [1] | 17 (20.7%) | | ≤6 [1] | 18 (13.5%) | | ≤6 [1] | 12 (11.6%) |
| 7(3+4) [2] | 23 (28.0%) | | 7(3+4) [2] | 22 (16.5%) | | 7(3+4) [2] | 13 (12.6%) |
| 7(4+3) [3] | 17 (20.7%) | | 7(4+3) [3] | 24 (18.0%) | | 7(4+3) [3] | 17 (16.5%) |
| 8 [4] | 9 (11.0%) | | 8 [4] | 22 (16.5%) | | 8 [4] | 18 (17.5%) |
| 9-10 [5] | 15 (18.3%) | | 9-10 [5] | 44 (33.2%) | | 9-10 [5] | 40 (38.8%) |
| Missing | 1 (1.3%) | | Missing | 3 (2.3%) | | Missing | 3 (3.0%) |
| Percent of positive biopsy cores (>34%) | | | Percent of positive biopsy cores (>34%) | | | Percent of positive biopsy cores (>34%) | |
| Positive | 44 (53.7%) | | Positive | 87 (65.4%) | | Positive | 76 (73.8%) |
| Negative | 37 (45.1%) | | Negative | 42 (31.6%) | | Negative | 24 (23.3%) |
| Missing | 1 (1.2%) | | Missing | 4 (3.0%) | | Missing | 3 (2.9%) |
| Clinical T | | | Metastasis Status | | | Metastasis Status | |
| T1c | 11 (13.4%) | | Positive | 54 (40.6%) | | Positive | 58 (56.3%) |
| T2a,T2b,T2c | 41 (50.0%) | | Negative | 79 (59.4%) | | Negative | 45 (43.7%) |
| T3a | 14 (17.1%) | | Metastatic PCa Volume | | | Metastatic PCa Volume | |
| T4 | 15 (18.3%) | | No Metastasis | 79 (59.4%) | | No Metastasis | 45(43.7%) |
| Missing | 1 (1.2%) | | Low | 26 (19.6%) | | Low | 28 (27.2%) |
| Primary Treatment | | | High | 28 (21.0%) | | High | 30 (29.1%) |
| RP | 62 (75.6%) | | Clinical T | | | Clinical T | |
| PRTX | 20 (24.2%) | | T1c | 12 (9.0%) | | T1c | 7 (6.8%) |
| HLA-A genotype | | | T2a,T2b,T2c | 34 (25.6%) | | T2a,T2b,T2c | 18 (17.5%) |
| HLA-A\*02:01+HLA-A\*24:02- | 23 (28.1%) | | T3a | 16 (12.0%) | | T3a | 12 (11.6%) |
| HLA-A\*24:02+ | 37 (45.1%) | | T4 | 69 (51.9%) | | T4 | 64 (62.1%) |
| HLA-A\*02:01-HLA-A\*24:02- | 22 (26.8%) | | Missing | 2 (1.5%) | | Missing | 2 (2.0%) |
| | | | Primary Treatment | | | Primary Treatment | |
| | | | RP | 52 (39.1%) | | RP | 31 (30.0%) |
| | | | PRTX | 23 (17.3%) | | PRTX | 11 (10.8%) |
| | | | ADT | 58 (43.6%) | | ADT | 61 (59.2%) |
| | | | HLA-A genotype | | | HLA-A genotype | |
| | | | HLA-A\*02:01+HLA-A\*24:02- | 43 (32.4%) | | HLA-A\*02:01+HLA-A\*24:02- | 33 (32.0%) |
| | | | HLA-A\*24:02+ | 49 (36.8%) | | HLA-A\*24:02+ | 38 (36.9%) |
| | | | HLA-A\*02:01-HLA-A\*24:02- | 41 (30.8%) | | HLA-A\*02:01-HLA-A\*24:02- | 32 (31.1%) |

## Slide 5
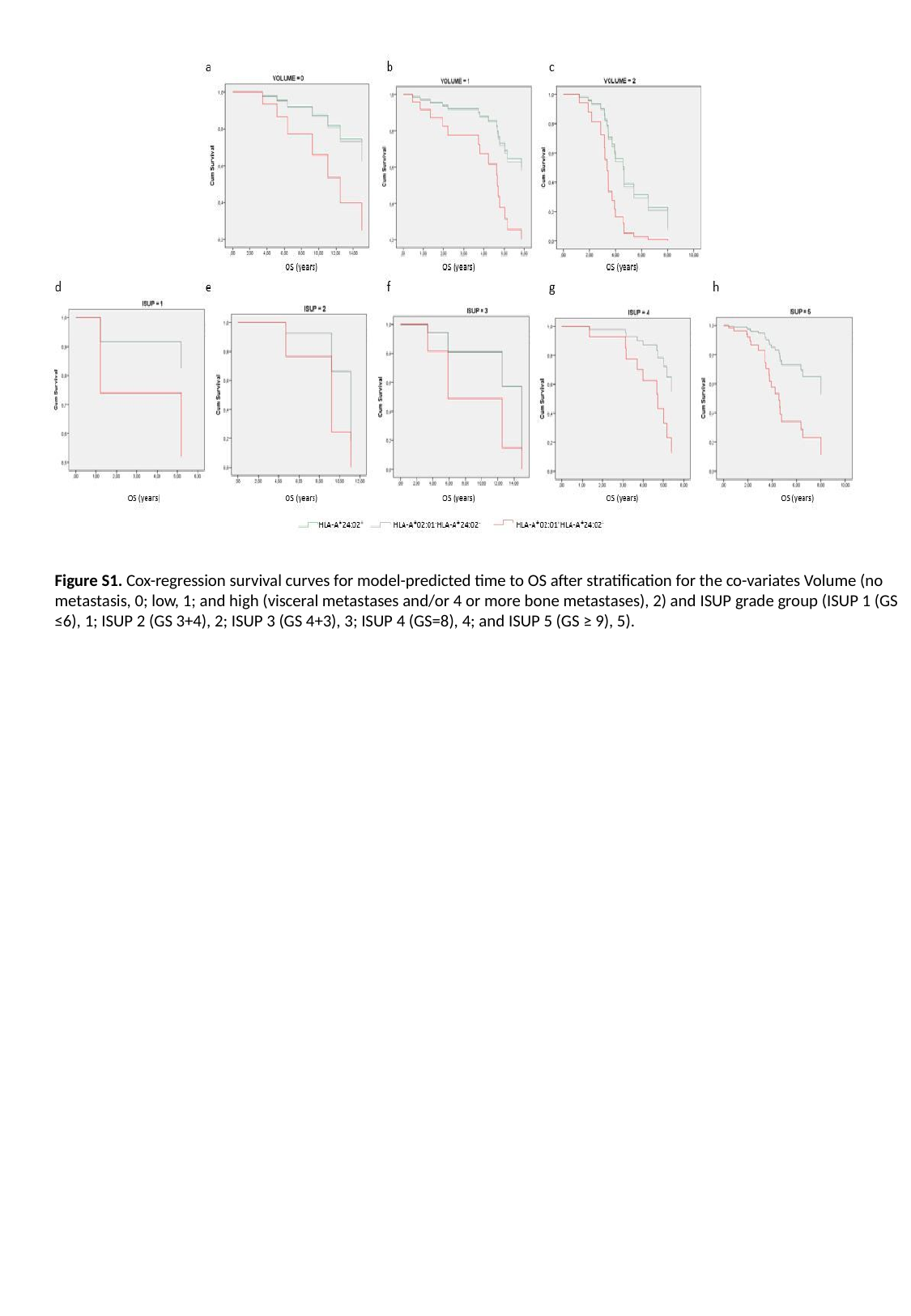

Figure S1. Cox-regression survival curves for model-predicted time to OS after stratification for the co-variates Volume (no metastasis, 0; low, 1; and high (visceral metastases and/or 4 or more bone metastases), 2) and ISUP grade group (ISUP 1 (GS ≤6), 1; ISUP 2 (GS 3+4), 2; ISUP 3 (GS 4+3), 3; ISUP 4 (GS=8), 4; and ISUP 5 (GS ≥ 9), 5).

## Slide 6
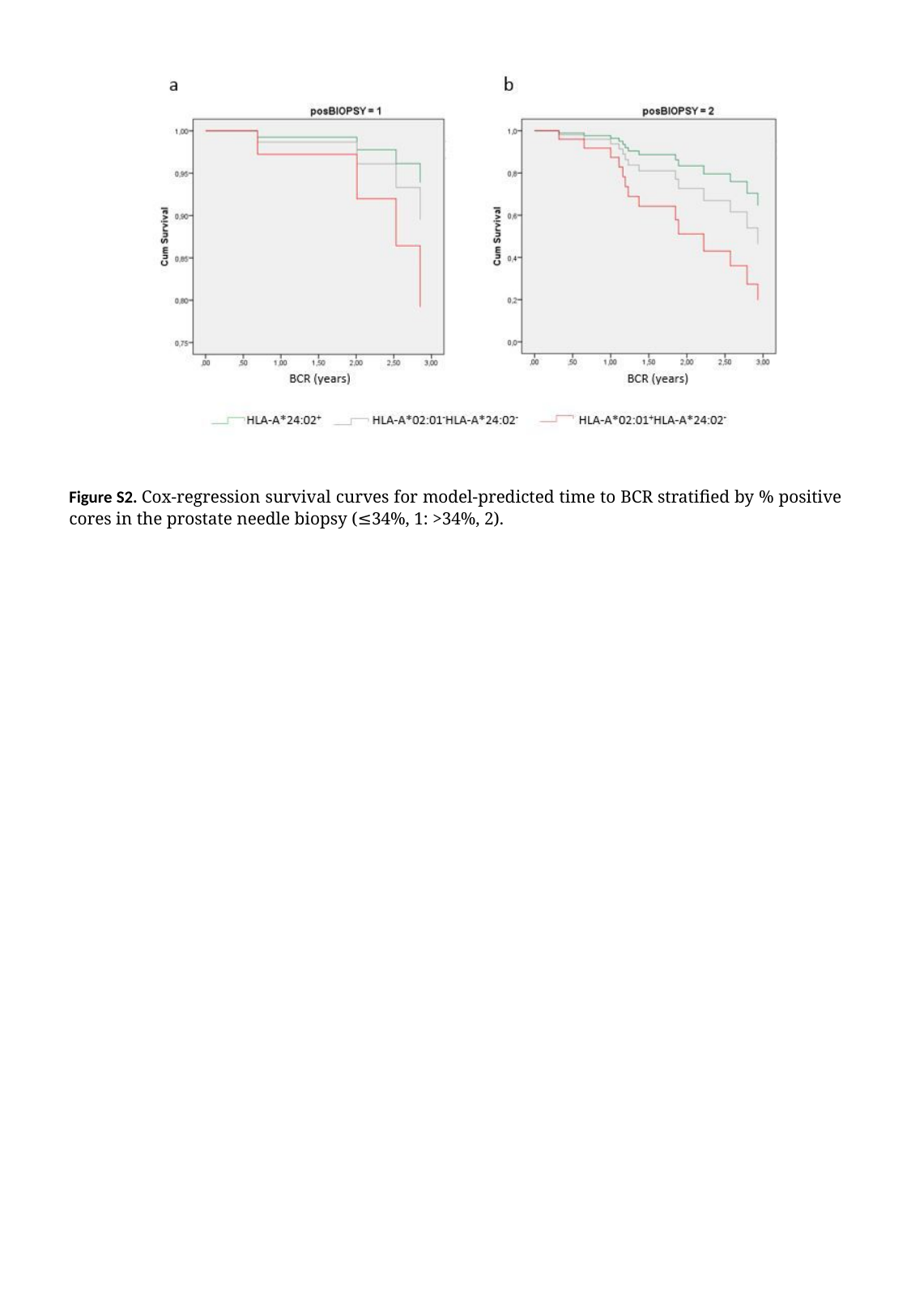

Figure S2. Cox-regression survival curves for model-predicted time to BCR stratified by % positive cores in the prostate needle biopsy (≤34%, 1: >34%, 2).

## Slide 7
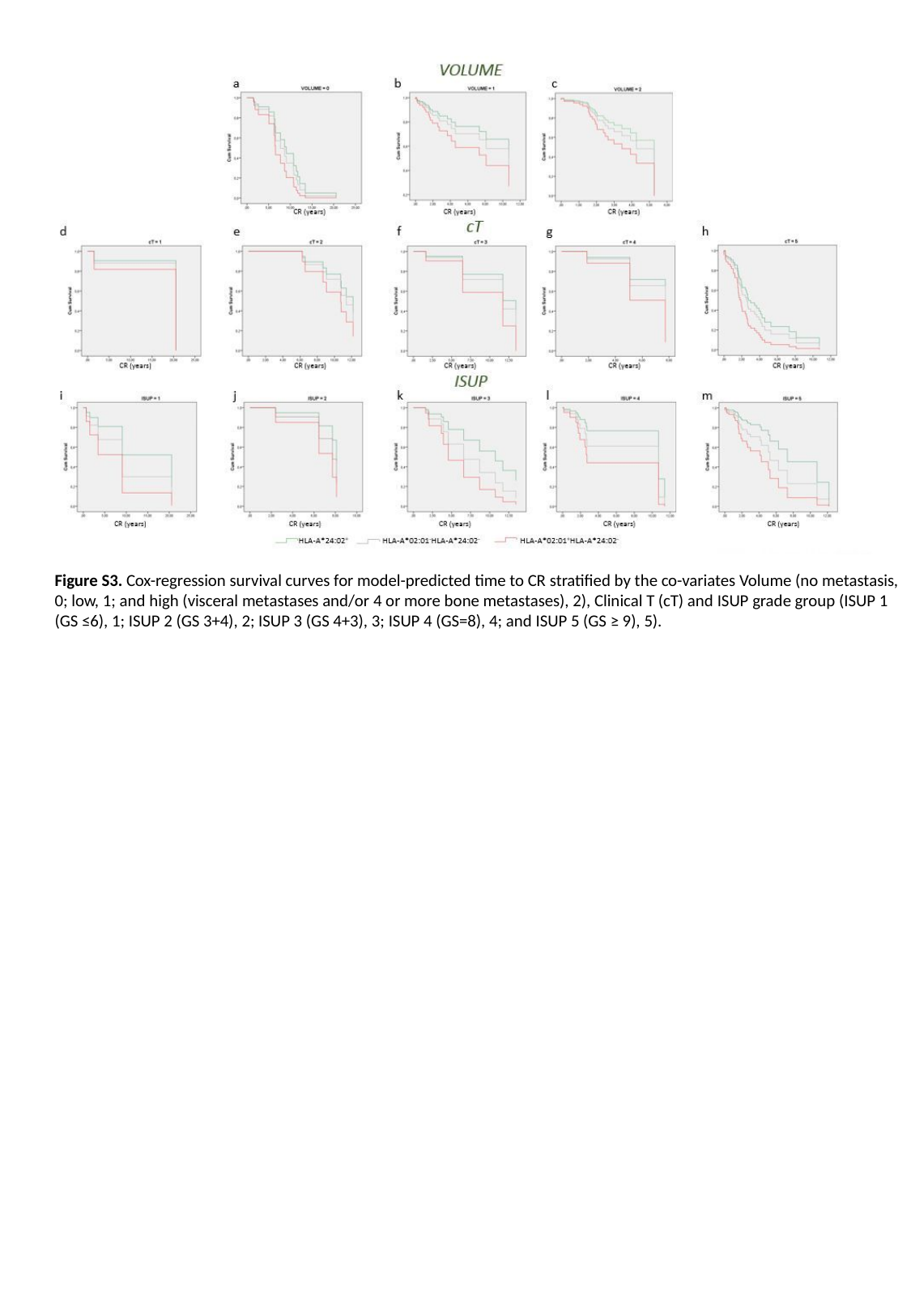

Figure S3. Cox-regression survival curves for model-predicted time to CR stratified by the co-variates Volume (no metastasis, 0; low, 1; and high (visceral metastases and/or 4 or more bone metastases), 2), Clinical T (cT) and ISUP grade group (ISUP 1 (GS ≤6), 1; ISUP 2 (GS 3+4), 2; ISUP 3 (GS 4+3), 3; ISUP 4 (GS=8), 4; and ISUP 5 (GS ≥ 9), 5).
